# Myeloid reprogramming generates myeloid-derived activating cells to potentiate radiotherapy and suppress metastasis

**DOI:** 10.1016/j.ccell.2026.08.017

**Published:** 2026-09-17

**Authors:** Fei Ji, Lei Zheng, Dapeng Chen, Peng Xia, Zhongyu Zou, Wei Liu, Jason Bugno, Emile Z. Naccasha, Andras Piffko, Chuangyu Wen, Kangdi Li, Liping Xu, Sean Z. Luo, Xiaona Huang, Jiaai Wang, Chengwei Ju, Bei Liu, Yiyi Ji, Han Li, Steven J. Chmura, Sean P. Pitroda, Hua Laura Liang, Chuan He, Ralph R Weichselbaum

**Affiliations:** 1 Department of Radiation and Cellular Oncology,; 2 The Ludwig Center for Metastasis Research, University of Chicago, Chicago, IL, 60637, USA; 3 Present address: Department of Breast Cancer, Cancer Center, Guangdong Provincial People’s Hospital, Southern Medical University, Guangzhou, Guangdong, 510080, China.; 4 Department of Chemistry, The University of Chicago, Chicago, IL 60637, USA; 5 Howard Hughes Medical Institute, Chicago, IL 60637, USA; 6 Committee on Clinical Pharmacology and Pharmacogenomics, University of Chicago, Chicago IL, USA; 7 Pritzker School of Medicine, The University of Chicago, Chicago, IL, USA; 8 Present address: Department of Neurosurgery, University Medical Center Hamburg-Eppendorf, Hamburg, Germany; 9 Present address: Department of Radiation Oncology, The First Affiliated Hospital of Nanjing Medical University, Nanjing, 210029, China; 10 Department of Biochemistry and Molecular Biology, University of Chicago, Chicago, IL 60637, USA.

**Keywords:** myeloid reprogramming, myeloid-derived suppressor cells (MDSCs), myeloid-derived activating cells (MDACs), radiotherapy, T cell cytotoxicity, immunotherapy

## Abstract

Radiation induces immunosuppressive myeloid cells that drive therapeutic resistance and metastasis. We identify TET2 as a radiation-induced regulator of myeloid-derived suppressor cells whose expression correlates with poor outcome in advanced lung cancer patients receiving radio-immunotherapy. Following radiotherapy, myeloid-specific *Tet2* deletion suppresses tumor progression and redirects monocyte differentiation toward antigen-presenting myeloid-derived activating cells (MDACs), which augment antitumor T cell responses; spatiotemporal mapping shows this fate program initiates rapidly in the bone marrow. Mechanistically, a radiation-responsive p53–TET2 axis decreases m^5^C on chromatin-associated RNAs (caRNAs), enforcing immunosuppressive chromatin compaction and silencing interferon signaling, whereas *Tet2* loss restores chromatin accessibility and enhances CD8^+^ T cell cytotoxicity. Pharmacological TET2 inhibition recapitulates this phenotype and augments radiotherapy and PD-L1 blockade to control primary and metastatic tumors. These findings define MDACs as an immunogenic myeloid subset and establish myeloid reprogramming as a strategy to improve radiotherapy–immunotherapy outcomes.

## Introduction

Radiotherapy (RT), a mainstay of cancer treatment given to 50–60% of patients, elicits complex immunomodulatory effects^1^. While mediating tumor cell death via DNA damage, RT simultaneously expands immunosuppressive populations including myeloid-derived suppressor cells (MDSCs)^2–5^. These cells employ multiple mechanisms to suppress innate and adaptive immunity^6–9^ with radiation enhancing their recruitment and immunosuppressive activity within tumors^10–13^. Clinically RT-induced MDSC expansion correlates with reduced survival and increased metastatic progression^14–17^, highlighting the need to overcome this axis.

The role of TET2 in cancer immunity is complex and context-dependent. Clonal hematopoiesis of indeterminate potential (CHIP) caused by TET2 inactivation is associated with worse outcomes in non-small cell lung cancer (NSCLC)^18^, yet paradoxically enhances immunotherapy response through increased IFN signaling in macrophages^19,20^. Beyond its DNA demethylase activity driving myeloid malignancies^21–26^and promoting immunosuppression via *Arg1*^27^. TET2 also functions as an RNA demethylase, oxidizing m^5^C on chromatin-associated RNAs (caRNAs) to recruit MBD6–BAP1 complexes for chromatin remodeling^28–36^, marking a transition from DNA-centric to RNA-inclusive paradigms. However, how this dual-substrate specificity regulates myeloid plasticity in irradiated tumors remains unexplored.

Here, we uncover a spatiotemporal epigenetic program in which radiation activates a TET2–m^5^C switch. Radiation causes TET2-dependent systemic programming of myeloid cells, with the bone marrow as the earliest site of lineage bifurcation. Radiation-induced TET2 oxidizes m^5^C on caRNAs, establishing a repressive chromatin state that silences interferon responses and enforces immunosuppressive MDSC differentiation, whereas *Tet2* ablation recruits MBD6–BAP1 complexes to erase these marks, restore chromatin accessibility, and drive differentiation toward immunostimulatory myeloid-derived activating cells (MDACs). These findings identify caRNA m^5^C as a direct regulator of chromatin topology and myeloid fate, establishing myeloid reprogramming as a strategy to overcome treatment resistance and metastasis.

## Results

### TET2 upregulation in myeloid cells impairs radiotherapy efficacy in tumors

Given previous reports of immune modulation by TET2, we examined its potential role in radiotherapy response using matched pre- and post-treatment peripheral blood mononuclear cells(PBMC) samples from patients with stage IV NSCLC enrolled in a phase I/II trial combining nivolumab and ipilimumab with sequential or concurrent multisite stereotactic body radiation therapy(SBRT)(NCT03223155; n = 33)^37^. M-MDSCs (Lineage^−^CD33^+^CD14^+^) exhibited the highest baseline TET2 expression relative to other immune cells (Figures 1A–1B). Radiation significantly increased TET2 in M-MDSCs (Figures 1C–1D), with a more pronounced increase in non-responders than responders (*p* = 0.0072 and *p* = 0.0532, respectively; Figure 1D). Similar trends were observed when patients were stratified by sequential or concurrent treatment, with significant increases in non-responders but not responders (Figures S1A–S1B). These findings suggest that radiotherapy-associated TET2 induction is not restricted to non-responders but more pronounced in this group, consistent with a general myeloid stress response whose magnitude may be associated with impaired therapeutic response.

We next evaluated whether myeloid *Tet2* deletion affects radiation response. Lyz^Cre^*Tet2*^fl/fl^ (*Tet2* cKO) mice and control *Tet2*^fl/fl^ littermates (WT) bearing MC38 tumors received a single dose of 20 Gy irradiation (IR)^10^. With equal tumor cell inocula, *Tet2* cKO mice exhibited slower post-IR tumor growth than WT controls (Figure S1C). After adjusting inoculation doses (*Tet2* cKO: 2×10^6^ vs WT: 1×10^6^ cells) to achieve comparable tumor volumes at irradiation, *Tet2* cKO mice showed superior radiation response and prolonged survival versus WT+IR controls, with *Tet2* cKO+IR exhibiting significant additional tumor suppression over *Tet2* deletion alone (Figures 1E and S1D). These findings were validated in B16-F1-OVA-ZsGreen melanoma and Lewis lung carcinoma (LLC) models (Figures 1F–1G and S1E).

In the spontaneous LLC metastasis model, combined *Tet2* deletion and radiotherapy further reduced pulmonary metastatic burden (nodule number and area) compared to either treatment alone (Figures 1H–1I and S1F). To exclude confounding by primary tumor burden, tumors were resected 7 days post-irradiation when no significant size differences were observed; *Tet2* deficiency remained associated with reduced lung metastasis, an effect further enhanced in the *Tet2* cKO+IR group (Figures S1G–S1H), demonstrating that myeloid *Tet2* deletion enhances both local and distant anti-tumor effects of irradiation.

Flow cytometric profiling showed that irradiation significantly expanded CD11b^+^Ly6C^+^ monocytic MDSCs (mMDSCs) in WT tumors (Figures 1J and S1J–S1L), an effect abrogated by myeloid-specific *Tet2* deletion, with similar findings in lung metastases (Figure S1I). CD11b^+^Ly6C^+^ cells sorted from *Tet2* cKO tumors and co-cultured with CD8^+^ T cells supported higher T cell proliferation than WT controls, indicating attenuated immunosuppressive capacity (Figure 1K). These findings established TET2 as a regulator of mMDSC recruitment and function in the irradiated tumor microenvironment.

### Cellular and molecular dissection of TET2-dependent immunosuppressive myeloid remodeling

To characterize myeloid dynamics at single-cell resolution, we performed scRNA-seq of CD45^+^ immune cells from MC38 tumors of WT or *Tet2* cKO mice with or without irradiation, identifying 21 clusters across seven major immune lineages (Figures S2A–S2B). Myeloid-focused analysis (Figure 2A) revealed that irradiation increased monocyte abundance in WT but not *Tet2* cKO tumors (Figure 2B), with sub-clustering identifying 21 unique myeloid populations (Figures 2C–2E). In WT control tumors, cluster 7 (Macro_Itga9), enriched for migration/differentiation genes (*Itga9*, *Itga6*, *Cebpa*) and the tolerance marker *Ctla-2b* (Figures S2C–S2D), predominated. Following irradiation, WT tumors instead showed expansion of two immunosuppressive populations: cluster 4_0 (Macro_Slfn4), marked by suppressive genes (*Slfn4*, *Ly6c2*, *Cd274*) and reduced antigen-presentation genes (*H2-Aa*, *H2-Ab1*) (Figure S2E); and cluster 3 (Macro_Hp), characterized by *Hp*, *Gsr*, *Foxp1*, and *Cd244a* (Figures S2J–S2K).

By contrast, *Tet2* cKO tumors exhibited a distinct myeloid landscape with expansion of immune-activating populations, including cluster 0_0 (Macro_Itgax), cluster 6 (Macro_Pltp), cluster 5_0 (Mono_Chst11), cluster 5_1 (Mono_Inhba), and cluster 1_0 (Macro_Cxcl3) (Figures S2F–S2I,S2L). Cluster 0_0 was enriched for antigen-presentation and phagocytic genes (*H2-Aa*, *H2-Ab1*, *H2-Eb1*, *H2-DMb1*, and *Itgax*) (Figure S2F), while monocyte subsets displayed robust inflammatory signatures: cluster 5_0 enriched for *Cxcl10*, *Ifi203*, *Il1b*, and *Cxcr4* (Figure S2G), cluster 5_1 for *Inhba*, *Il1b*, *Cxcl3*, *Clec4d*, and *Clec4e* (Figure S2H), and cluster 1_0 for *Ccl3*, *Inhba*, *Nos2*, and *Clec4d* (Figure S2L), further reinforcing the inflammatory program.

To define how *Tet2* regulates myeloid differentiation under irradiation, we performed trajectory reconstruction using diffusion mapping, pseudotime ordering, and Slingshot (Figures S3A–S3B and 2F–2G). Pseudotime analysis revealed a radiation-induced differentiation arrest in WT monocytes, evidenced by predominant retention at early pseudotime following irradiation (Figures 2H and S3C), whereas *Tet2*-deficient monocytes displayed robust differentiation progression resembling unirradiated controls regardless of irradiation (Figure 2I), indicating that *Tet2* loss overrides the radiation-imposed developmental blockade.

Cluster 3 occupied the earliest pseudotime position across all trajectory inference methods and served as the common transitional root state (Figure S3D), from which trajectory analysis revealed a clear bifurcation into two distinct fate programs (Figure 2J). Trajectory 1 progressed through clusters 3 → 5_0 → 5_1 → 1_0, culminating in an inflammatory terminal state marked by elevated *Inhba*, *Clec4d*, *Il7r*, and *Tnfrsf1b* (Figures 2J and S2L), while Trajectory 2 progressed from 3 → 0_1, corresponding to the radiation-associated suppressive program in WT tumors (Figures 2J and S2L). The distribution of these trajectories was genotype dependent: *Tet2* cKO+IR samples were enriched along the proinflammatory branch (Trajectory 1), whereas WT+IR samples occupied the suppressive branch (Trajectory 2) (Figure 2K), indicating that *Tet2* deficiency redirects monocyte differentiation toward proinflammatory fates under radiation. This was also confirmed by latent time and RNA velocity analysis (Figures S3E–S3I).

Comparative transcriptomic analysis revealed distinct gene expression programs between the two trajectories: Trajectory 1 showed increasing proinflammatory mediators (e.g., *Inhba*, *Cxcl2*, *Il7r*), whereas Trajectory 2 showed suppression of these genes and upregulation of immunosuppressive factors (e.g., *Arg1*, *Il1r2*) (Figures 2L and S3J), with pathway analysis confirming enrichment of IRF3-mediated type I interferon signaling in Trajectory 1 (Figure 2M). These data suggest a cytokine-dependent fate bifurcation model: elevated IFN-I signaling in *Tet2* cKO+IR conditions maintains monocytes in a proinflammatory state, whereas low IFN-I signaling in WT+IR tumors promotes immunosuppressive differentiation (Figure 2N).

### Radiation-activated TET2 establishes a caRNA m^5^C-gated chromatin switch to enforce myeloid immunosuppression

To investigate how TET2 regulates monocyte differentiation after radiation, we examined TET2 dynamics in irradiated tumors and myeloid cells. ScRNA-seq of CD45^+^ cells from MC38 tumors showed *Tet2* upregulation in irradiated monocytes, particularly clusters 3 and 5_1 (Figure 3A), confirmed in tumor-derived MDSCs (CD11b^+^Ly6C^+^), beginning day 1 after irradiation (Figures 3B–3C). Radiation also directly induced TET2 in bone marrow(BM)-derived monocytes dose- and time-dependently, with 8 Gy producing the strongest induction (Figures 3D–3F). RNA-seq pathway analysis identified “transcriptional regulation by p53” as a top enriched pathway (Figure 3G): p53 increased in parallel with TET2 in both BM-derived and tumor-infiltrating MDSCs (Figures 3I–3J), and *Trp53* knockdown abrogated radiation-induced TET2 expression (Figures 3K–3L). ChIP-qPCR confirmed p53 binding to the *Tet2* promoter ~1.5–2.0 kb upstream of the transcription start site (Figures 3H and 3M). These results establish that radiation activates the p53-TET2 axis to modulate m-MDSC function.

Since previous research has reported that TET2 is a demethylase of DNA and RNA^26–32^, we investigated two potential mediators of TET2 function: (1) CXXC5, which directs TET2 to enhancer DNA 5mC oxidation, and (2) the m5C reader MBD6, which binds RNA m^5^C^29^. Using antisense oligonucleotides (ASOs) to knock down *Mbd6* and *Cxxc5* in *Tet2*-deficient monocytes (Figures S4A–S4C), we found that the enhanced T cell proliferation and activation seen in *Tet2* KO monocytes versus wild-type controls was completely abrogated by *Mbd6* knockdown but not *Cxxc5* knockdown (Figures S4D–S4F), indicating that TET2-mediated monocyte functional alteration relies primarily on MBD6-dependent RNA m^5^C recognition. *Tet2*-deficient monocytes also exhibited genome-wide increases in chromatin accessibility and transcriptional activity, as shown by EU incorporation, DNase-TUNEL, and ATAC-seq (Figures S4G–S4K), establishing TET2 as an epigenetic regulator that maintains chromatin compaction to restrain transcription—a phenotype consistent with TET2’s RNA m^5^C oxidation role rather than its canonical DNA demethylation function^38,39^. ASO-mediated knockdown of *Mbd6* (RNA m^5^C reader), but not *Cxxc5* (binding protein to DNA), reversed *Tet2* deficiency-associated chromatin decompaction (Figures S4L–4M), revealing MBD6 and RNA m^5^C as critical downstream mediators of TET2 in an RNA-dependent regulatory axis governing chromatin dynamics and transcriptional output.

The RNA-dependent chromatin regulation by TET2 is known to be mediated through chromatin-associated RNAs (caRNAs)^40–44^. We performed caRNA-seq integrated with chromatin accessibility data in irradiated monocytes to test whether TET2 alters caRNA landscapes and observed a concomitant reduction in caRNA and caRNA m^5^C levels after radiation (Figures S5A–S5B and S5D–S5E). Genetic deletion of *Tet2* reversed both the IR-mediated suppression of caRNA and loss of m^5^C modification (Figures S5C–S5E), indicating caRNA m^5^C oxidation by TET2 in WT cells after radiation.

It has been reported that radiation induces initial chromatin relaxation (for DNA repair) followed by compaction^45–48^. Image-based assays (EU, TUNEL) showed reduced radiation-induced chromatin compaction in *Tet2*-deficient cells (Figures 4A–4B), confirmed by ATAC-seq (Figures 4C–4D); these changes correlated with caRNA expression and transcriptional activity (Figures 4E–4F), and regions losing accessibility after IR were enriched for IFN-I response genes (Figures 4G–4I). IR downregulated IRF3 expression, whereas *Tet2* deficiency restored IRF3 levels and reversed IR-mediated IFN-β suppression (Figures 4J–4M), and IFNAR1 blockade abrogated the enhanced antitumor response to IR in *Tet2* cKO mice (Figure 4N), establishing IRF3-mediated IFN-I signaling as the critical link between *Tet2* ablation and improved radiation response. Together, these data indicate that radiation induces p53-mediated TET2 upregulation, leading to m^5^C oxidation on caRNAs, chromatin compaction, and repression of interferon responses that enforce immunosuppressive M-MDSC differentiation (Figure 4O).

### The caRNA-MBD6-H2AK119ub Axis Activates IRF3-mediated Interferon Signaling by Remodeling Chromatin

We next investigated whether caRNAs mediate the effects of TET2 on IFN signaling. Consistent with increased global chromatin accessibility in irradiated *Tet2* KO monocytes (Figure 4D), ATAC-seq revealed increased accessibility at the *Irf3* promoter (Figure 5A), and caRNA-seq identified elevated expression of the retrotransposon-derived caRNA *Rmer21b* at the *Irf3* promoter under *Tet2* cKO+IR conditions (Figure 5B), with increased m^5^C methylation on *Rmer21b* (m^5^C-MeRIP-qPCR; Figure 5C). *Rmer21b*-targeting ASOs reduced *Irf3* chromatin accessibility and decreased IRF3 and IFN-β expression in irradiated *Tet2* KO monocytes (Figures 5D–5E and S5F), indicating that caRNA *Rmer21b* promotes IRF3-mediated IFN-β production through local chromatin modulation.

To validate the functional role of *Rmer21b* in *Tet2* KO-mediated antitumor efficacy, we adoptively transferred WT, *Tet2* KO, or *Rmer21b*-silenced *Tet2* KO BM-myeloid cells into Lyz^Cre^CSF1R^Cre^-DTR mice following DT-mediated depletion of endogenous myeloid cells. Transfer of *Tet2* KO myeloid cells enhanced radiation response, an effect abrogated by *Rmer21b* silencing (Figure 5F), confirming its functional importance in vivo. Given that MBD6 has been identified as a reader of caRNA m^5^C^29^, we further confirmed MBD6 binding to m^5^C-marked *Rmer21b* by MBD6-CLIP-PCR (Figures S5G and 5G). *Mbd6* knockdown in irradiated *Tet2* KO BM-monocytes phenocopied *Rmer21b* silencing, reducing *Irf3* chromatin accessibility and IRF3/IFN-β expression (Figures 5D, 5H, S5H), and adoptive transfer of *Mbd6*-silenced *Tet2* KO monocytes abolished the enhanced radiation response conferred by *Tet2* KO cells (Figure 5I), identifying *Rmer21b* and MBD6 as critical effectors of this caRNA m^5^C-dependent epitranscriptomic–epigenetic axis.

Our analysis of histone profiles identified H2AK119ub, a canonical repressive histone marker that regulates chromatin accessibility, as the most dynamically altered modification in *Tet2*-deficient monocytes following irradiation (Figures 5J–5L): it accumulated in wild-type cells after radiation but was significantly reduced in irradiated *Tet2* KO monocytes. In irradiated monocytes, H2AK119ub also correlated inversely with chromatin accessibility (Figure 5A) and global transcriptional activity (Figure 5M), suggesting it plays a pivotal role in the TET2-dependent epigenetic response to radiation by coordinately regulating chromatin architecture and gene expression.

H2AK119ub is reported to be removed by polycomb repressive deubiquitylase (PR-DUB) complexes^49,50^, with BAP1 being the catalytic component and MBD6 as one of the partner proteins^29,51,52^. We hypothesized that MBD6 is required for H2AK119ub removal. H2AK119ub cut&tag in *Tet2* KO or *Mbd6* knockdown *Tet2* KO irradiated monocytes showed an increased H2AK119ub signal in *Mbd6* KD *Tet2* KO monocytes compared to *Tet2* KO (Figure 5N), correlating with increased protein levels (Figure S5I); depletion of the caRNA *Rmer21b* similarly elevated H2AK119ub (Figures 5N and S5J). These results support a model in which radiation triggers p53-mediated TET2 upregulation, decreasing caRNA m^5^C modification to drive chromatin compaction and suppress type I interferon responses, whereas *Tet2* deficiency activates the IRF3/IFN-I pathway through a caRNA-MBD6-H2AK119ub axis that orchestrates radiation-induced chromatin remodeling (Figure 5O), ultimately reprogramming tumor-associated myeloid cells toward a proinflammatory phenotype in the irradiated tumor microenvironment.

### TET2-dependent divergent myeloid fates emerge from the bone marrow after irradiation

Having established the molecular mechanism by which TET2 regulates caRNA m^5^C oxidation, H2AK119ub deposition, chromatin accessibility, and downstream interferon signaling, we next sought to determine the spatio-temporal mechanism of myeloid fate programming induced by radiation in the context of *Tet2* deficiency. To address this, we performed a compartment-specific time-course analysis across bone marrow, peripheral blood, and tumor tissues at 0, 1, 2, and 3 days post-irradiation of primary tumors in both WT and *Tet2* cKO mice.

Following irradiation, WT and *Tet2* cKO mice exhibited comparable early tumor damage and systemic DAMP release, reflected by similar kinetics of γH2AX accumulation and plasma HMGB1 elevation (Figure S6A), indicating that the upstream radiation-induced stress signal remains intact in both genotypes. Irradiated tumor-conditioned medium similarly induced an early p53 response in bone marrow monocytes from both hosts (Figure S6B), confirming that the initial DNA damage/repair response is TET2-independent.

The most rapid and pronounced divergence emerged in the bone marrow compartment. In WT mice, bone marrow monocytes displayed the fastest induction of p-p53 and TET2, detectable by day 1 post-irradiation and progressively increasing through day 3 (Figure S6C–S6D), accompanied by accumulation of the repressive H2AK119ub and progressive suppression of *Irf3* and *Ifnb1* (Figures S6E–S6G), consistent with a chromatin program favoring suppressive myeloid differentiation. In contrast, *Tet2*-deficient bone marrow monocytes preserved upstream p53 activation but lacked TET2 induction, showed impaired H2AK119ub accumulation, and maintained persistent IRF3–IFN-I signaling throughout (Figures S6D–S6G). These findings indicate that myeloid fate specification is determined in the bone marrow rapidly following radiation.

Peripheral blood monocytes exhibited a similar but delayed divergence, suggesting progressive propagation of this program during systemic trafficking, while tumor-infiltrating monocytes displayed the slowest, most fully executed differences, consistent with terminal differentiation into immunosuppressive MDSCs in WT hosts or pro-inflammatory monocytes in *Tet2*-deficient hosts.

Together, these data support a model in which local radiation triggers systemic stress signaling and p53 activation, with the bone marrow as the earliest bifurcation site for lineage commitment: under WT conditions, a p53-TET2-dependent trajectory drives suppressive MDSC differentiation, whereas under *Tet2* deficiency the same stress is redirected into a pro-inflammatory monocyte lineage, with full functional execution occurring only after these primed cells migrate into the tumor microenvironment (Figure S6H).

### Pro-inflammatory myeloid-derived activating cells (MDACs) prime CD8^+^ T cells through antigen presentation

We showed above that the IRF3/IFN-I pathway was activated in irradiated *Tet2* KO monocytes, yielding enhanced proinflammatory gene signatures: a distinct immunostimulatory phenotype marked by upregulation of genes for antigen recognition (*Clec4d*, *Clec4e*) and antigen presentation (*CD74*), inflammatory cytokines (*Il1a*, *Il1b*, *Tnf*), and interferon-stimulated genes (Figures S2G–S2H and S7A). These inflammatory monocytes highly express the interferon-regulated surface marker *Itgal*^53^ (Figures S2G–S2H), and T cell chemoattractants *Cxcl9* and *Cxcl10* (Figures 6A–6B and S7B–S7D). ITGAL is a core component of the lymphocyte function-associated antigen-1 (LFA-1), mediating stable monocytes-T cells adhesion fundamental to antigen presentation and T cell activation. This adhesive interface supports immune synapse formation and downstream T cell responses^54–56^. Flow analysis confirmed that ITGAL^+^ monocyte frequency was significantly increased in irradiated *Tet2* cKO tumors (Figures 6C–6D). Together, these immunostimulatory features and ITGAL expression define a proinflammatory myeloid-derived activating cell (MDAC, CD11b^+^Ly6C^+^ITGAL^+^) driven by IFN-I signaling - a fundamental reprogramming of myeloid function under combined *Tet2* deficiency and radiation.

We next investigated T cell activation by immunoprofiling tumor-infiltrating lymphocytes. *Tet2* cKO tumors exhibited significantly higher CD8+ T cell proportion and activation than WT tumors (Figures 6E–6F and S7E–S7F), defining distinct microenvironmental states-immunologically active (*Tet2* cKO+IR) versus immunosuppressive (WT+IR). CD8+ T cells were required for enhanced tumor control in *Tet2* cKO hosts, as their depletion abolished the augmented response (Figure 6G), establishing CD8+ T cell reinvigoration as a key pathway underlying the synergistic anti-tumor effects of *Tet2* ablation and radiotherapy.

To identify the cellular drivers of CD8^+^ T cell activation in irradiated tumors, we integrated spatial mapping with functional validation. Immunofluorescence showed MDACs positioned significantly closer to CD8^+^ T cells than dendritic cells (DCs), suggesting preferential MDAC-CD8^+^ T cell interactions over conventional DCs in irradiated *Tet2* cKO tumors (Figures 6H–6I). Among inflammatory Ly6C^+^ monocytes in the *Tet2* cKO+IR group, the ITGAL^+^ subset (MDACs) accounted for predominant antigen-presenting activity (Figure 6J), and isolated Ly6C^+^ITGAL^+^ cells robustly promoted naïve CD8^+^ T cell proliferation and IFN-γ production (Figures 6K–6M), supporting a direct role for MDACs in local antitumor T cell activation. Transfer of CD11b^+^Ly6C^+^ITGAL^+^ cells significantly suppressed tumor growth versus control and CD11b^+^Ly6C^+^ITGAL⁻ populations, providing direct in vivo evidence of antitumor activity (Figure 6N).

To further validate the role of inflammatory monocytes in CD8^+^ T cell priming, we generated Lyz^Cre^*Tet2*^fl/fl^-CD11c-DTR mice, in which *Tet2* is deleted in myeloid cells and CD11c^+^ dendritic cells can be selectively ablated by diphtheria toxin (Figure S7G). DC depletion did not alter CD8^+^ T cell activation (Figure S7H) or tumor response to irradiation (Figure S7I) versus the *Tet2* cKO+IR group, indicating that intratumoral CD8^+^ T cell priming is mediated primarily by *Tet2*-deficient MDACs rather than conventional DCs, and establishing MDACs as necessary and sufficient for restoring antitumor CD8^+^ T cell immunity (Figure S7J).

To assess the clinical relevance of this program, we further analyzed patients with TET2-mutant clonal hematopoiesis (CH)^20^ and TCGA data, and found that the pro-inflammatory transcriptional program associated with Cluster 5_0, Cluster 5_1, and Trajectory 1 in *Tet2*-deficient myeloid cells was significantly enriched in TET2-mutant CH patients by GSEA (Figures S7K–S7M). The human MDAC signature (CD33, ITGAM, ITGAL) was likewise associated with improved overall survival across multiple cancer cohorts, including radiotherapy-treated patients (Figures 6O and S7N).

### Pharmacological TET2 inhibition potentiates radiotherapy and immunotherapy

To test whether pharmacological TET2 inhibition phenocopies *Tet2* genetic deletion, we treated tumor-bearing mice with a TET inhibitor (TET-inhibitor-C35) (Figure S8A)^57,58^. Consistent with *Tet2* cKO mice, TET inhibition suppressed tumor growth in both MC38 and B16 models and further enhanced antitumor efficacy of IR over IR alone (Figures 7A–7B and S8B–S8C), and in the LLC spontaneous metastasis model, TET inhibition combined with IR reduced both number and size of lung metastases versus IR alone (Figures 7C–7E). These data suggest TET inhibition enhances the antitumor efficacy of irradiation, though this additional benefit was modest and the dominant therapeutic effect was driven by TET inhibition itself.

TET inhibitor treatment had no significant effect on tumor cell viability across tested concentrations (Figures S8D–S8F), suggesting host immunity was involved. Irradiation increased CD11b^+^Ly6C^+^Ly6G⁻ myeloid infiltration in WT tumors, an expansion abolished by TET inhibition (Figure 7F), which also markedly enhanced CD8^+^ T cell responses, with increased GzmB^+^ and IFN-γ^+^ CD8^+^ T cells, and reduced exhaustion markers PD-1, TIM-3, and LAG-3 (Figures 7G–7H and S8G–S8J), consistent with irradiated *Tet2* cKO mice. In MC38 model, TET inhibition improved tumor control and anti-PD-L1 efficacy; irradiation added further benefit, though TET inhibition remained the dominant effect (Figure 7J).

TET2 deficiency in hematopoietic stem cells is linked to myeloid malignancies. Although the Bana group^59^ demonstrated that pre-leukemic myeloproliferation arises exclusively in *Tet2*^f/f^*Vav*^Cre^mice, but not in *Tet2*^f/f^*Lyz*^Cre^ mice, consistent with the latter, TET inhibitor treatment did not increase CD11b^+^Gr1^+^ pre-leukemic myeloproliferation markers^59^ or splenic weights across all treated groups (Figures S8K–S8P). These findings indicate that short-term, low-dose TET inhibition does not induce leukemogenesis and instead enhances radio-immunotherapy efficacy synergistically.

## Discussion

Increased TET2 expression in MDSCs from patients receiving radio–immunotherapy was associated with poor responses, implicating TET2 as a regulator of therapy resistance. Myeloid-specific *Tet2* deletion synergized with irradiation to suppress primary tumor growth and metastasis in preclinical models by reprogramming monocyte toward a pro-inflammatory/immunostimulatory state - myeloid derived activating cells (MDACs) marked by co-expression of CD11b, Ly6C, and ITGAL. Mechanistically, *Tet2* deficiency preserves caRNA m^5^C, enabling MBD6-(PR-DUB) complexes to erase repressive H2AK119ub marks, restore chromatin accessibility, and reinstates IFN-I responses. This epigenetic reprogramming enhanced radiation’s therapeutic effects by improving CD8^+^ T cell cytotoxicity and suppressing metastatic spread, revealing a targetable interface between RNA epitranscriptomics and chromatin regulation in cancer immunity.

Our findings place this mechanism within a spatiotemporal framework of radiation-induced myeloid reprogramming, in which the bone marrow is the earliest site of lineage bifurcation. Although upstream radiation-induced stress signals are comparable, divergence emerges in epigenetic execution: under WT conditions, activation of the p53–TET2 axis enforces a repressive chromatin program promoting immunosuppressive MDSC differentiation, whereas *Tet2* deficiency redirects bone marrow monocytes toward an MDAC fate. This program is propagated during systemic trafficking and amplified within the tumor microenvironment, where MDACs acquire antigen-presenting capacity and drive CD8^+^ T cell activation.

These findings establish competing epitranscriptomic programs governing myeloid function: an immunosuppressive circuit maintained by radiation-TET2-m^5^C oxidation versus an immunostimulatory circuit activated by m^5^C preservation. This framework extends current models of stress-induced myelopoiesis^60^ by introducing an epigenetic bifurcation mechanism in which TET2 acts as a molecular switch between suppressive MDSC and immunostimulatory MDAC fates. Thus, tumor resistance is not static but a dynamic, reversible process governed by epigenetic lineage programming. Therapeutically, targeting this m^5^C-gated chromatin switch offers a strategy to reprogram myeloid fate and overcome radiotherapy/immunotherapy resistance. The efficacy of TET inhibition in combination with irradiation and immune checkpoint blockade in preclinical models highlights the potential to manipulate these epitranscriptomic checkpoints without inducing hematopoietic toxicity^19,61^. By redirecting myeloid differentiation toward MDACs, this approach enables simultaneous control of local tumor growth and metastasis.

In conclusion, our study defines a p53–TET2–caRNA epigenetic axis linking radiation-induced stress signaling to chromatin remodeling and systemic myeloid lineage differentiation, providing a mechanistic framework for radiotherapy resistance and a roadmap for restoring antitumor immunity through myeloid reprogramming.

### Limitations of the study

Several limitations should be noted. First, our mechanistic studies relied on murine models and ex vivo or in vitro-cultured myeloid cells, which may not capture human tumor-associated MDSC heterogeneity. Although our CD3/CD28-based suppression assays support reduced suppressive activity of irradiated myeloid cells upon *Tet2* loss, validation using patient-derived MDSCs and MDACs will be important. Second, spatial and functional imaging studies are needed to define MDAC interactions with T cells within tumors. Third, pharmacological TET inhibition improved tumor control and radiotherapy efficacy, but its additive benefit with irradiation was modest in the models tested. Future studies combining other modalities and humanized tumor models are warranted.

### RESOURCE AVAILABILITY

#### Lead contact

Further information and requests for resources should be directed to Ralph Weichselbaum (rweichselbaum@bsd.uchicago.edu).

#### Materials availability

This study did not generate new, unique reagents.

#### Data and code availability

- All data generated or analyzed during this study are included in this published article. All raw sequencing data have been uploaded to GEO and SRA database, available under the accession number GSE304925 and PRJNA1305028.
- This paper does not report original code.
- All other data of this study are available from the corresponding author on reasonable request.

## STAR METHODS

### EXPERIMENT MODEL AND STUDY PARTICIPANT DETAILS

#### Patients samples

Peripheral blood mononuclear cells (PBMCs) were obtained from patients with metastatic non-small cell lung cancer (NSCLC) enrolled in our phase I COSINR trial (NCT03223155) receiving sequential or concurrent SBRT and immune checkpoint blockade therapy (Ipilimumab and Nivolumab). Paired samples were collected pre-treatment and post-SBRT completion under protocols approved by the University of Chicago Biological Sciences Division Institutional Review Board (IRB-17-0547), with written informed consent obtained from all participants in accordance with the Declaration of Helsinki.

#### Cancer cell lines

The MC38 murine colon adenocarcinoma, LLC Lewis lung carcinoma, and B16F10 melanoma cell lines were obtained from ATCC (Manassas, VA). The B16-SIINFEKL(OT-I)-ZsGreen (B16-OZ) cell line was generated and maintained in our laboratory. All cell lines were cultured in Dulbecco’s Modified Eagle Medium (DMEM) (Corning, Manassas, VA) supplemented with 10% heat-inactivated fetal bovine serum (FBS) (Sigma, St Louis, MO) and 1% penicillin-streptomycin (10,000 U/mL penicillin, 10 mg/mL streptomycin), and maintained at 37°C in a humidified 5% CO2 atmosphere. All cell lines were routinely tested and confirmed negative for mycoplasma contamination. ATCC-derived cell lines were authenticated by the provider and used within limited passages after thawing. The B16-SIINFEKL(OT-I)-ZsGreen cell line was generated and maintained in our laboratory and was validated by antigen/ZsGreen expression before use.

#### Mice

All mice were housed and used according to the animal experimental guidelines set by the Institute of Animal Care and Use Committee of The University of Chicago. All animals were maintained in pathogen-free conditions and cared for in accordance with the International Association for Assessment and Accreditation of Laboratory Animal Care policies and certification. *Tet2*^flox/flox^ mice, *Lyz*^Cre^ *Tet2*^flox/flox^ mice, C57BL/6 mice were purchased from The Jackson Laboratory. Male and female mice aged eight to ten weeks were used in the experiments.

### METHOD DETAILS

#### Tumor growth and treatment

MC38, LLC, and B16 tumor cells (1×10^6^) were implanted subcutaneously into the right flanks of WT or *Tet2* cKO mice. Once tumors reached ~100 mm^3^ (length × width × height × 0.5), mice received a single 20 Gy dose of localized ionizing radiation (IR) or sham treatment. Lung metastasis arising from LLC tumours was assessed at the end point, 30 days after tumor implantation.*Tet2* cKO mice exhibited significantly delayed tumor growth compared to WT controls (Figure S1D). To ensure comparable baseline tumor volumes at the time of irradiation, *Tet2* cKO mice received a higher initial tumor cell inoculum (2 × 10^6^ cells) versus wild-type controls (1 × 10^6^ cells) in designated experiments.

For anti–PD-L1 therapy, mice received 200μg of anti–PD-L1 antibody intraperitoneally, twice weekly for four doses. For TET inhibitor treatment, 10 μM of the compound was administered intravenously every other day for five doses, with appropriate vehicle controls. In DT-mediated monocytes depletion and adoptive transfer experiments, Lyz^cre^CSF1R-DTR mice were injected intraperitoneally with DT (50 ng/mouse), followed the next day by tumor-localized IR and transfer of WT/*Tet2* KO/*Tet2* KO *Rmer21b*-ASO–treated/*Tet2* KO *Mbd6*-ASO–treated monocytes. A second DT dose was given on day 5. For CD8^+^ T cell depletion, 200 μg of anti-CD8α antibody was administered intraperitoneally one day prior to treatment initiation and continued twice weekly thereafter.Tumor growth was monitored biweekly for 3–4 weeks. Each experiments were independently repeated 3 times with consistent results.

#### Flow cytometry

Tumors and lymph nodes were harvested and processed to generate single-cell suspensions. Tumor tissues were minced and enzymatically digested using 1 mg/ml collagenase I/IV (C4-22-1G, Sigma-Aldrich) and 200 μg/ml DNase I (04536282001, Millipore Sigma) at 37°C for 45 min, while lymph node cells were isolated by mechanical dissociation through 70 μm strainers. Cell suspensions were filtered (70 μm), washed twice in PBS-based staining buffer (2% FBS, 0.5 mM EDTA). Dead cells were stained with Zombie-NIR(BioLegend, 423105). Cells were blocked with anti-FcγR antibodies(2.4G2, CP025, BioXcell, 1:100) for 10 min. Surface staining was performed using fluorochrome-conjugated antibodies (1:200 dilution) for 30 min at 4°C in the dark. For intracellular targets, cells were fixed/permeabilized (BD Cytofix/Cytoperm solution, 554722, Fisher Scientific) prior to antibody staining. For intracellular staining of IFN-γ, cells were stimulated with Cell Activation Cocktail with Brefeldin A(Biolegend,423303) for 6–8 h and followed by intracellular staining using BD Cytofix/Cytoperm intracellular staining kit. For TET2 intracellular staining, cells were fixed, washed twice with diluted Permeabilization Buffer, then incubated overnight at 4°C with anti-TET2 primary antibody. After washing, cells were stained with Alexa Fluor 647-conjugated anti-rabbit IgG secondary antibody (Life Technologies, 60 min, 4°C, dark). All samples were analyzed on a BD Fortessa flow cytometer, with data processed using FlowJo v10 software.

#### Histology

Tissues were fixed with 4% paraformaldehyde for a minimum of 24 hours and sent to the university core facility for embedding and processing. The slides were stained using Leica Bond RX automated stainer. The protocol named as “No Post Primary 1h Bond DAB Refine” was used. Epitope retrieval solution I (Leica Biosystems, AR9961) was used for heat treatment for 20 minutes. Slides were scanned using CRi Panoramic SCAN 40x Whole Slide Scanner and analyzed by QuPath (v0.1.2). One section or two sections at a distance of 200 μm were analyzed per lung at experimental endpoint.

Volumetric microscopy of mouse tumors was performed as previously described^17,69^. Briefly, excised tumors were fixed in 4% paraformaldehyde for 24 h, paraffin-embedded, and sectioned at The University of Chicago HTRC core facility. Following deparaffinization in xylene and rehydration through graded ethanol series, antigen retrieval was conducted in citrate buffer (90°C, 45 min). Tissue sections were blocked with 5% BSA and immunostained using anti-CD8 (BioLegend, 53–6.7; 1:100), anti-FSCN1 (Santa Cruz, 55-k2; 1:50), and anti-ITGAL (BioLegend, H155–78; 1:200) antibodies. Stained sections were mounted in Mowiol and imaged on a Leica TCS SP8 confocal microscope equipped with a 20×/0.75 NA objective. Whole-tumor 3D image stacks were acquired with 2 μm z-spacing (minimum 20 μm depth), processed using Leica LIGHTNING deconvolution (v3.5.7), and analyzed with Imaris 9.9 software for 3D surface reconstruction of individual cells.

#### ELISPOT assay

CD8+ T cells were isolated from MC38-OVA tumors three days post-irradiation (2–4×10^5^ cells/condition) and stimulated ± 1 μg/ml SIINFEKL peptide for 48–72 hr. In parallel, naïve CD11c+ DCs were pulsed with irradiated tumor cells (6 hr), then co-cultured with CD8+ T cells (48–72 hr). IFN-γ production was quantified by ELISPOT (BDB551873, BD Biosciences) using manufacturer’s protocols, with spot analysis performed on an ELISPOT plate reader.

#### Cytokine detection

MC38 tumors were harvested from WT or *Tet2* cKO mice three days post-IR, homogenized in PBS with protease inhibitors (1:100,78442, Thermo Fisher Scientific,), and centrifuged (12,000 rpm, 10 min) to obtain supernatant. Cytokine concentrations were quantified using the LEGENDplex Mouse Inflammation Panel (13-plex; 740446, BioLegend) on V-bottom plates, with acquisition on a BD Fortessa flow cytometer and data analysis using LEGENDplex v8.0 software (BioLegend).

#### BM-monocyte induction and isolation

Bone marrow was harvested from wild-type (WT) and *Tet2* cKO mice to prepare single-cell suspensions, hereafter referred to as fresh bone marrow cells. These cells were cultured in RPMI-1640 medium supplemented with 10% heat-inactivated fetal bovine serum (FBS) and 20 ng/ml recombinant mouse GM-CSF (carrier-free,576306, BioLegend). On day 2, fresh medium containing GM-CSF was added to maintain cytokine levels. On day 4, CD11b^+^Ly6C^+^ bone marrow-derived monocytes were isolated from the cultured bone marrow cells through flow sorting.

#### T cell suppression assay

Tumor-infiltrating or bone marrow-derived MDSCs were co-cultured with naïve splenic CD8+ T cells isolated by EasySep Mouse CD8^+^ T Cell Isolation Kit(19853, STEMCELL) at a 4:1 (T cell:MDSC) ratio. CD8^+^ T cells were pre-labeled with CellTrace CFSE (C34554, Invitrogen) and stimulated with anti-CD3/CD28 beads(11453D, Thermo Fisher) prior to co-culture. Proliferation was assessed by flow cytometric analysis of CFSE dilution.

#### Knockdown in BM-monocyte

SiRNA targeting mouse *Trp53* (sc-29436, Santa Cruz,)was transfected into bone marrow derived MDSCs by Lipofectamine^™^ RNAiMAX Transfection Reagent (13778150, Invitrogen) according to manufacturer’s protocol. The sequences of AUMsilence ASOs(Antisense oligonucleotides, ASOs) targeting *Mbd6*(TCAGAGGGCACCAGGTTGGAT), CaRNA-LTR-*Rmer21b*(TGTAAGTGTTTGTGCTTGGAT) and *Cxxc5*(AAGAGTAGTGAGAGAGTGGTC) were transfected into BM-monocyte according to manufacturer’s(AUM BioTech, LLC) protocol. Two days after the transfection, the cells were collected. The knockdown efficiency was detected by qPCR.

#### Quantitative real-time polymerase chain reaction (qRT-PCR)

Total RNA was extracted by using RNeasy Plus Mini Kit(Qiagen). cDNA was then synthesized by using High-Capacity cDNA Reverse Transcription Kit(4368814, Applied Biosystems). RT-qPCR using SYBR Green PCR Master Mix(4309155, Applied Biosystems) was performed in QuantStudio 3 according to manufacturer’s instruction. The specific mRNA primers for RT-qPCR are as follows: *Tet2* (Forward: AGAGAAGACAATCGAGAAGTCGG, Reverse:CCTTCCGTACTCCCAAACTCAT);*Mbd6*(Forward:ATGGAACGGTCCCCAAGAAG, Reverse:GCCACGACGAGACTTTCTGAC);*IFNβ* (Forward:GCACTGGGTGGAATGAGACT, Reverse:AGTGGAGAGCAGTTGAGGACA);*Rmer21b*(Forward:TTCTCCCTTCAGAGCTGCAT, Reverse:TGTAAGTGTTTGTGCTTGGATAGG); *Cxxc5*(Forward: GCCAAAAGGCAGGA GGAACAGA, Reverse:TGATACCGCTCTTGTTCCGACG); *Cxcl9*(Forward:CCT AGTGATAAGGAATGCACGATG, Reverse:CTAGGCAGGTTTGATCTCCGTTC); *Cxcl10*(Forward:ATCATCCCTGCGAGCCTATCCT, Reverse:GACCTTTTTTGGCTAAACGCTTTC-3). GAPDH was chosen as an endogenous control. Gene expression was calculated by using the 2-ΔΔCt method, fold difference = 2^−(ΔCtA −ΔCtB)^, where Ct represents cycle threshold.

#### Bulk mRNA-seq

Ly6C^+^BM-monocytes were generated from WT and *Tet2* cKO mice as described in ‘BM-monocyte induction and isolation’. Cells were either irradiated (8 Gy) or maintained as untreated controls. Two hours post-irradiation, total RNA was extracted using TRIzol reagent (15596026, Invitrogen) following the manufacturer’s protocol. Poly(A)+ mRNA was enriched using oligo(dT) magnetic beads, and stranded RNA-seq libraries were prepared using the SMARTer Stranded Total RNA-Seq Kit v2 - Pico Input Mammalian (634411, TaKaRa Bio) and quality-checked by Bioanalyzer (Agilent Technologies) prior to paired-end sequencing (2 × 150 bp) on an Illumina NovaSeq 6000 system (Illumina) with dual-indexing at the University of Chicago Genomics Core.

Raw sequencing reads were trimmed to remove adapter sequences and low-quality bases using Cutadapt (v4.8)1. The cleaned reads were then aligned to the Mus musculus reference genome (mm10) and transcriptome, along with External RNA Control Consortium (ERCC) spike-in sequences (Thermo Fisher Scientific, when applicable), using STAR aligner (v2.7.9a)^70^. Gene annotation files (M23) were downloaded from the GENCODE database (https://www.gencodegenes.org/)^71^. For chromatin-associated RNA (caRNA) analysis, regulatory regions were defined according to criteria described in a previous study^40^. Gene-level quantification for both GENCODE-annotated genes and chromatin-associated RNAs (caRNAs) was performed using featureCounts (v2.0.3)^72^. Factors of unwanted variation estimated by RUVSeq^73^, using ERCC spike-ins as negative controls, were used to normalize the expression matrix. Differential gene expression analysis was conducted using the DESeq2 package (v1.28.0)^74^. Genes with an P value < 0.05 were considered significantly differentially expressed. Gene set enrichment analysis was performed using GSEA^75^ and GSEApy^76^ for pathway enrichment and visualization.

#### Chromatin-associated RNA-seq(caRNA-seq)

Ly6C^+^ BM-monocytes were generated from WT and *Tet2* cKO mice as described in ‘BM-monocyte induction and isolation’. Cells were either irradiated (8 Gy) or left untreated as controls. Two hours post-irradiation, chromatin-associated RNAs (caRNAs) were extracted using our previously established protocol^29^. RNA samples were then subjected to ribosomal RNA depletion using the RiboMinus Eukaryote System v2 (A15026, Invitrogen) followed by size selection with the RNA Clean & Concentrator-5 kit (R1013, Zymo Research) according to manufacturer’s instructions. High-quality caRNA libraries were prepared using the SMARTer Stranded Total RNA-Seq Kit v2 - Pico Input Mammalian (634411, TaKaRa Bio), with all steps performed as specified in the product manual. Finally, paired-end sequencing was carried out on the Illumina NovaSeq 6000 platform (Illumina) at the University of Chicago Genomics Core.

#### EU-based nascent RNA labeling analysis

Nascent RNA was labeled by incubating cells with 1 mM 5-ethynyl uridine (EU) for 60 min at 37°C, followed by detection using Click-iT^™^ RNA Alexa Fluor^™^ 488 Imaging Kit according to manufacturer’s protocol. Cells were washed with 0.5% BSA/PBS (3,000 × g, 3 min, RT) between steps, except during the Click-iT^®^ reaction rinse which used the specified buffer.

#### DNase I–TUNEL assay

To quantitatively assess chromatin accessibility, treated monocytes were subjected to DNase I-TUNEL assay following established protocols with modifications. Briefly, ASO-transfected cells were fixed with 4% PFA, permeabilized with 0.1% Triton X-100, and treated with 1 U/ml DNase I (EN0521, Thermo Fisher) for 5 min at 37°C before labeling with DeadEnd Fluorometric TUNEL System (G3250, Promega). propriate controls including DNase I-treated positive controls and rTdT-omitted negative controls were processed in parallel. High-resolution confocal imaging was performed on a Leica SP8 microscope (63× oil objective) with standardized acquisition parameters.

#### Assay for Transposase-Accessible Chromatin using sequencing(ATAC-seq)

ATAC-seq was performed on 100,000 Ly6C^+^ BM-monocytes isolated from wild-type (WT) and *Tet2* conditional knockout (*Tet2* cKO) mice, either irradiated (8 Gy) or left untreated as controls. Two hours post-irradiation, cells were processed using the ATAC-seq kit (53150, Active Motif) according to the manufacturer’s protocol. Each replicate included an equal amount of Drosophila spike-in DNA (53154, Active Motif) for normalization.

Cells were permeabilized in a buffer containing 0.1% Tween-20 and 0.01% digitonin (supplied with the kit), and accessible chromatin regions were tagmented using a pre-assembled Tn5 transposome complex. Following genomic DNA extraction, libraries were prepared via PCR amplification and pooled for sequencing on an Illumina NovaSeq 6000 platform at the University of Chicago Genomics Core.

Sequencing reads were first trimmed using Cutadapt (v4.8) to remove adapter sequences and low-quality bases. Cleaned reads were aligned to the *Mus musculus* reference genome (mm10) using Bowtie2 (v2.4.4)^77^, and only uniquely mapped reads were retained. Reads aligned to the mitochondrial genome and PCR duplicates were removed. MACS3 (v3.0.1)^66^ was used to identify ATAC-seq peaks. Peaks overlapping with ENCODE blacklist regions were excluded to minimize potential artifacts. Peaks consistently detected in at least two biological replicates were merged using BEDTools (v2.31.0)^78^ for downstream analyses. The chromatin accessibility (ATAC) were normalized by considering both sequencing depth and spike-in Drosophila melanogaster chromatin. To identify differentially accessible chromatin regions, read counts over merged peaks were quantified using featureCounts (v2.0.3)^72^. Differential accessibility analysis was then performed using DESeq2 (v1.28.0)^74^. Functional enrichment analysis of the differentially accessible regions was conducted using GSEA^76^.

#### Quantitative LC-MS/MS analysis of RNA modifications

For RNA modification profiling, ribodepleted RNA (75 ng) was sequentially digested with nuclease P1 (Sigma N8630; 20 mM ammonium acetate, pH 5.3, 42°C, 2 h) followed by FastAP thermosensitive alkaline phosphatase treatment (Thermo EF0651; 1× buffer, 37°C, 2 h). Digested samples were filtered (0.22 μm) and analyzed by LC-MS/MS (Agilent 6460) using a C18 reversed-phase column in positive electrospray ionization mode. RNA modifications were quantified via specific mass transitions (m5C: 258→126; hm5C: 274→142) against pure nucleoside standard curves, with three technical replicates per sample. Data were normalized to total nucleoside content and analyzed using Agilent MassHunter Quantitative Analysis software (v10.0).

#### m^5^C-RNA Immunoprecipitation and Sequencing

Spike-in RNA mixes (m^5^C-modified/unmodified) were prepared as previously described and added to chromatin-associated RNA prior to fragmentation. RNA was fragmented in 1× NEB fragmentation buffer (E6186A; 94°C, 4 min), with reactions halted by stop solution.

For immunoprecipitation, 4 μg of anti-m^5^C antibody (MAb-081-100, Diagenode) or control IgG (ab37355, Abcam) was conjugated to protein G beads (1003D, Invitrogen) in IP buffer (10 mM Tris-HCl pH 7.5, 150 mM NaCl, 0.05% Triton X-100, 1 mM spermidine) for 2 h at 4°C. Denatured caRNA (10 μg) from BM-monocyte of four treatment groups(WT/*Tet*2 KO with/without IR) was incubated overnight at 4°C with antibody-bound beads in the presence of SUPERaseIn RNase Inhibitor (AM2694, Invitrogen). After stringent washing, bound RNAs were eluted in buffer (5 mM Tris-HCl pH 7.5, 1 mM EDTA, 0.05% SDS, proteinase K; 50°C, 1 h), purified using Zymo RNA Clean & Concentrator-5(R1013, Zymo Research), and quantified by RT-qPCR.

For library preparation, immunoprecipitated RNAs were processed with the SMARTer Stranded Total RNA-Seq Kit v2 (634411, TaKaRa) and sequenced on an Illumina NovaSeq 6000 platform at the University of Chicago Genomics Core.

#### Western blot

Cells were lysed in RIPA buffer (89900, Thermo Fisher Scientific) supplemented with 1× Halt protease and phosphatase inhibitor cocktail (78441, Thermo Fisher Scientific). Protein concentrations were determined using Pierce^™^ BCA Protein Assay Kits (23227, Thermo Fisher Scientific). Equal amounts of total protein were denatured in 1× Laemmli loading buffer (1610747, Bio-Rad) at 90°C for 10 min, resolved on 4–12% NuPAGE Bis-Tris gels (NP0335BOX, Invitrogen), and transferred to PVDF membranes (88585, Thermo Fisher Scientific).

Membranes were blocked with 3% BSA (A7030, Millipore-Sigma) in TBST (Tris-buffered saline with 0.1% Tween-20) for 60 min at room temperature, followed by incubation with primary antibodies overnight at 4°C. After washing, membranes were probed with HRP-conjugated secondary antibodies for 1 h at room temperature. Protein bands were detected using SuperSignal West Dura chemiluminescent substrate (34075, Thermo Fisher Scientific) and imaged on a FluorChem R system (ProteinSimple).

#### Chromatin immunoprecipitation (ChIP) qPCR Assay

ChIP assays were performed using the Magna ChIP A/G Kit (17-10085, Millipore) following the manufacturer’s protocol. Briefly, 1×10^7^ Ly6c^+^ BM-monocytes were fixed with 1% formaldehyde for 10 min at room temperature to crosslink protein-DNA complexes. Cells were lysed and chromatin was sheared by sonication to obtain DNA fragments averaging 200–500 bp. Chromatin extracts were immunoprecipitated overnight at 4°C with 5 μg of target-specific antibody or control IgG, coupled to protein A/G magnetic beads. After extensive washing, protein-DNA complexes were eluted and crosslinks were reversed by incubation at 65°C for 4 h. DNA was purified using the kit’s purification columns and analyzed by quantitative PCR with primers specific to the *Tet2* promoter region.For quantification, 1% of the input chromatin was processed in parallel as an internal control. Enrichment was calculated using the percentage of input method: (100×2^((Input Ct - 6.64) – *Tet2* Ct)^), where Ct represents threshold cycle values. All qPCR reactions were performed in technical triplicates.

#### CUT&Tag analysis

Cleavage under targets and tagmentation (CUT&Tag) analysis was conducted using the CUT&Tag-IT Assay Kit (53160, Active Motif) following the manufacturer’s protocol. 100,000 Ly6C^+^ BM-monocytes isolated from WT and *Tet2* cKO mice, either irradiated (8 Gy) or left untreated as controls. Two hours post-irradiation, cells were washed with 1× wash buffer. The washed cells were conjugated to concanavalin A beads and permeabilized using a Digitonin-containing buffer before incubation with primary antibodies (anti-H2AK119ub or normal rabbit IgG). Preassembled protein A-Tn5 transposome complexes were employed for DNA tagmentation following secondary antibody conjugation. Equal amounts of Drosophila spike-in chromatin preps (53083, Active Motif) were added to each sample and subjected to the Tn5 tagmentation reaction. Tagged DNA was extracted by proteinase K digestion and amplified by PCR with indexed primers to construct DNA libraries. These DNA libraries were subjected to high-throughput sequencing using the NovaSeq 6000 platform at the University of Chicago Genomics Core.

High-quality reads were aligned to the *Mus musculus* reference genome (mm10) using Bowtie2 (v2.4.4)^77^ with the following parameters: --end-to-end --very-sensitive --no-mixed --no-discordant --phred33 --minins 10 --maxins 700 --dovetail −1. MACS3 (v3.0.1)^66^ was used for peak calling with the options: --nomodel --keep-dup auto -q 1e-4 --broad --broad-cutoff 0.01 -f BAMPE. Peak annotation was performed using the annotatePeaks.pl script from HOMER (v4.11)^79^. Signal visualization across genomic regions was carried out using IGV (v2.6.3)^80^. Signal intensities for H2AK119ub CUT&Tag were quantified using BEDTools^78^, and read counts were normalized to RPKM in 20-bp bins across defined genomic regions. The normalized signals were used to generate heatmaps with deepTools (v3.3.1)^81^, including those showing signal overlap between CUT&Tag and ATAC-seq datasets.

#### Quantitative analysis of RNA modification levels of Cross-linking and immunoprecipitation(CLIP) RNA

Radiated BM-monocytes from WT or *Tet2* cKO mice were washed twice with DPBS before UV cross-linking at 254 nm using a Stratalinker (Stratagene). The cells were then resuspended in three volumes of ice-cold CLIP lysis buffer (50 mM HEPES, pH 7.5, 150 mM KCl, 2 mM EDTA, 0.5% (v/v) NP-40, 0.5 mM DTT, 1× Halt protease and phosphatase inhibitor cocktail (78442, Thermo Fisher Scientific), and 1× RNaseOUT recombinant ribonuclease inhibitor (10777019, Invitrogen). The pellets were lysed by rotating at 4 °C for 15 min. The cell suspensions were subsequently sonicated using the Bioruptor system (Diagenode) with 30s on/30s off for five cycles. Lysates were cleared by centrifugation at 21,000g for 15 min at 4 °C in a benchtop centrifuge. The supernatants were incubated with MBD6-antibody (gifted from Prof. Lu Wang^82^)-conjugated protein G beads (Invitrogen, 1001D) overnight at 4 °C on an end-to-end rotor. Beads were washed extensively with 1 ml wash buffer (50 mM HEPES, pH 7.5, 300 mM KCl, 0.05% (v/v) NP-40, 1× Halt protease and phosphatase inhibitor cocktail, and 1× RNaseOUT recombinant ribonuclease inhibitor) at 4 °C five times. Input and IP samples were subsequently treated with proteinase K (25530049, Invitrogen) to release cross-linked RNA, which was recovered using TRIzol reagent (15596026, Invitrogen). Ribosomal RNA was then depleted using the RiboMinus Eukaryote System v2 (Invitrogen, A15026), followed by purification and size-selection with the RNA Clean & Concentrator-5 kit (R1013, Zymo Research). The recovered RNAs were then subjected to RT-PCR for downstream analysis.

#### Single-cell RNA-seq (scRNA) analysis

To profile the immune landscape of MC38 tumors, we performed single-cell RNA sequencing (scRNA-seq) on CD45^+^ immune cells isolated from wild-type (WT) and *Tet2* conditional knockout (*Tet2* cKO) mice, with or without prior irradiation (20 Gy). Tumors were pooled and dissociated into single-cell suspensions. Viable cells were labeled with Zombie NIR viability dye (30 min), followed by anti-mouse CD45 antibody staining (30 min). Zombie NIR^−^CD45^+^ single cells were FACS-sorted and processed for scRNA-seq library preparation using the Chromium Next GEM Single Cell 3’ GEM, Library & Gel Bead Kit v3.1 (1000128), following the manufacturer’s protocol. Libraries were sequenced on an Illumina HiSeq X Ten platform (University of Chicago Genomics Facility), with a target recovery of ~8,000 cells per library.

Raw single-cell RNA sequencing (scRNA-seq) data were processed using Cell Ranger (v7.2.0, 10X Genomics). Briefly, Illumina base call files (BCL) were demultiplexed into FASTQ format using cellranger mkfastq. Sequencing reads were then aligned to the mouse reference genome (mm10; GENCODE vM23/Ensembl 98, released July 2020 by 10X Genomics) and filtered for unique molecular identifiers (UMIs) using cellranger count. This pipeline generated a digital gene expression matrix, where each entry represents the UMI count for a given gene in an individual cell

Low-quality cells with UMIs beyond two standard deviation or having mitochondrial reads larger than 25% were discarded from further analyses.For scRNA-seq of mMDSC, as we applied Cell Hashing method using a series of oligo-tagged antibodies against ubiquitously expressed surface proteins with different barcodes to uniquely label cells from distinct samples, we demultiplex cells into different samples based on HTO enrichment by using HTODemux method in Seurat package (v4.4.0).

The processed whole gene expression matrix was then fed to Seurat (v4.4.0) for downstream analyses. Briefly, the UMI count matrix was normalized by using ‘SCTransformfunction’^83^. ‘Hormany’^84^ package was used to correct the batch effect among groups. Clustering analyses were performed using the first 30 principal components for constructing the shared nearest neighbor (SNN) graph by using ‘FindNeighbors’ function, and then Louvain clustering algorithm was used to group the cells into different clusters. Next, we applied scClassify (v1.2.0)^63^ for cell type classification based on cell types hierarchies constructed from reference datasets (E-MTAB-8832, CD45+ immune cells sorted from MC38 tumor-bearing C57BL/6 mice)^85^.

For lineage trajectory analyses, we specifically restricted the analysis to monocyte-derived populations. Trajectory inference was performed on CD45^+^CD11b^+^Ly6C^+^ cells and closely related monocyte-derived clusters, thereby excluding macrophage populations with transcriptional features consistent with tissue residency. In addition, clusters expressing canonical tissue-resident macrophage markers, including Timd4, Lyve1, and F13a1, were excluded from trajectory reconstruction to minimize lineage ambiguity and ensure accurate inference of radiation-induced monocyte fate transitions.

#### TCGA data analysis

TCGA data was acquired and analyzed in part using the Xena Platform^86^. For survival analysis, an MDAC signature score based on expression of ITGAM, ITGAL, CD33 was applied to the TCGA data. For Pan cancer, Lung cancer cohort’s gene expression was divided into high and low expression based on the median gene expression of all samples. Samples in the lowest and highest groups received a score of −1 and 1, respectively. Scores were added for each sample. Samples were filtered for patients receiving radiation therapy. Survival data from these groups were then compared and significance calculated using the ggsurvplot function in R.

#### Quantification and statistical analysis

To estimate the statistical significance of differences between two groups, we used an unpaired Student’s t-test to calculate two-tailed *p*-values. One-way analysis of variance (ANOVA) or two-way ANOVA with multiple comparison tests was performed when more than two groups were compared. Survival analysis was conducted using Kaplan-Meier curves and evaluated with log-rank Mantel-Cox tests. Error bars represent the standard error of the mean (SEM), unless otherwise noted. *p*-values are indicated in the figures. Statistical analyses were performed using GraphPad Prism (version 10.0).

## Supplementary Material

Supplementary final0812

Supplemental information:

Document S1: Figures S1–S8.

## Figures and Tables

**Figure 1:**
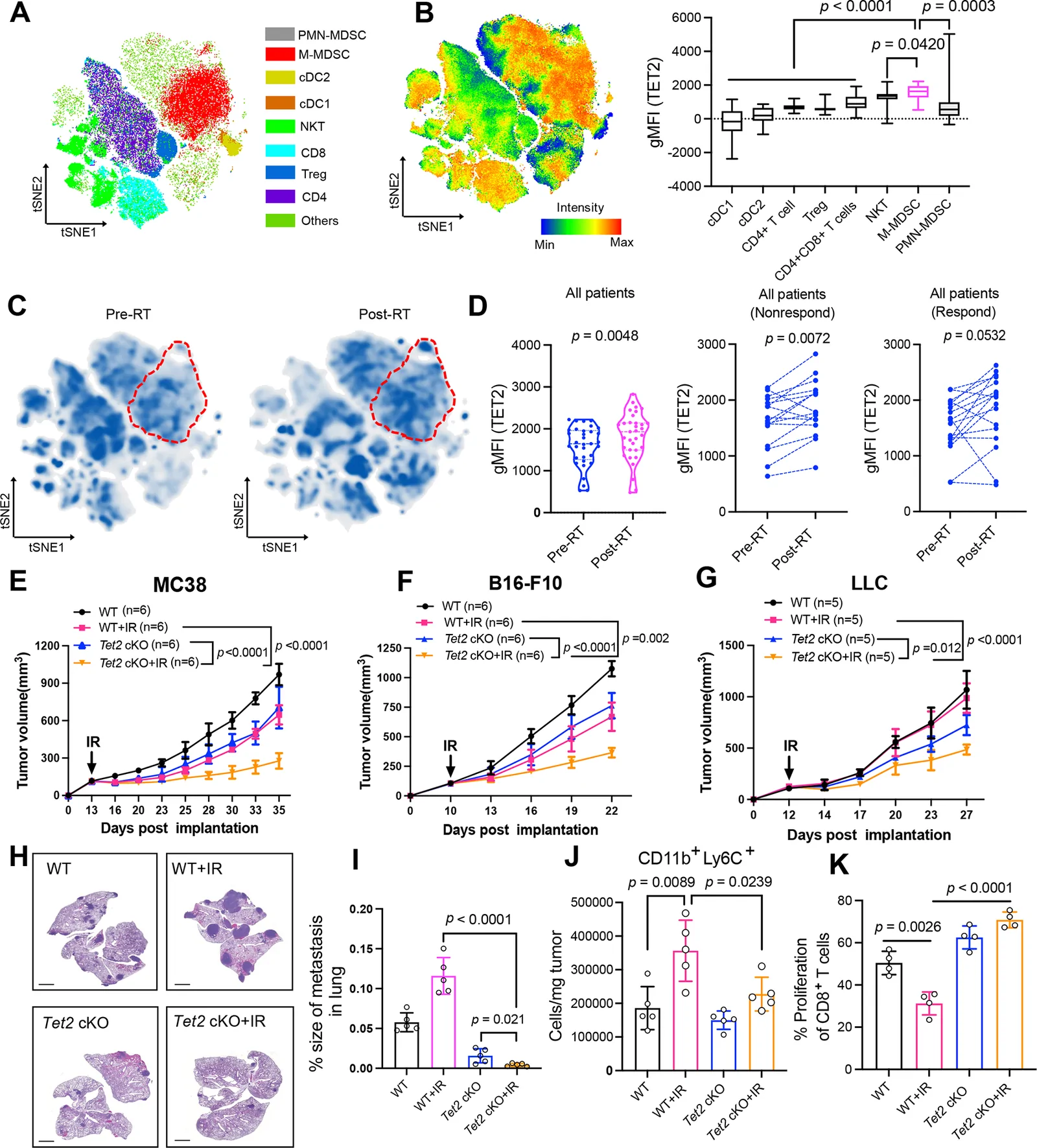
TET2 negatively impacts tumor response to radiotherapy. **(A)** t-SNE clustering of live CD45^+^ PBMCs of matched pre- and post-SBRT samples analyzed by Spectral flow cytometry (n = 33). **(B)** TET2 expression intensity before SBRT projected on t-SNE plots (left) and baseline TET2 gMFI in manually gated immune populations (right). Box plots show median, interquartile range, and min–max whiskers. One-way ANOVA with Bonferroni’s multiple comparisons test. **(C)** Density plots of t-SNE from PBMCs pre- and post-RT with M-MDSC population outlined. **(D)** gMFI of TET2 in M-MDSCs before and after radiotherapy. Left, all patients; middle, nonresponders; right, responders. Dots indicate patients. Dotted lines show median and interquartile range; dashed lines connect paired samples. Paired two-tailed Student’s t-test. **(E-G)** Tumor growth in WT and *Tet2* cKO mice bearing MC38 (E), B16-F10 (F), or LLC (G) tumors treated with or without local IR (20 Gy, single dose). Data are mean ± SD; n = 6 mice per group for MC38 and B16-F10, and n = 5 mice per group for LLC. Two-way ANOVA with correction for multiple comparisons. **(H)** Representative H&E stainings of lungs of flank LLC-tumor-bearing mice 30 days after tumor implantation. Four treatments are as indicated. Scale bar, 2 mm. **(I)** Lung metastases size (LLC) in mice 30 days after tumor implantation. mean size of the five largest metastases per lung area. Data are shown as mean ± SD;each dot represents one lung.One-way ANOVA with Bonferroni’s multiple comparison tests. **(J)** Tumor-infiltrating CD11b^+^Ly6C^+^ cell numbers in WT and *Tet2* cKO mice 3 days after IR. Data are mean ± SD; each dot represents one tumor (n = 5 per group). One-way ANOVA with Bonferroni’s multiple comparisons test. **(K)** Flow cytometry analysis of T cell proliferation in the suppressing assay with CD11b^+^Ly6C^+^ cells from tumors in WT or *Tet2* cKO mice three days after IR. Data are shown as mean ± SD;each dot represents a replicate.One-way ANOVA with Bonferroni’s multiple comparison tests.

**Figure 2.**
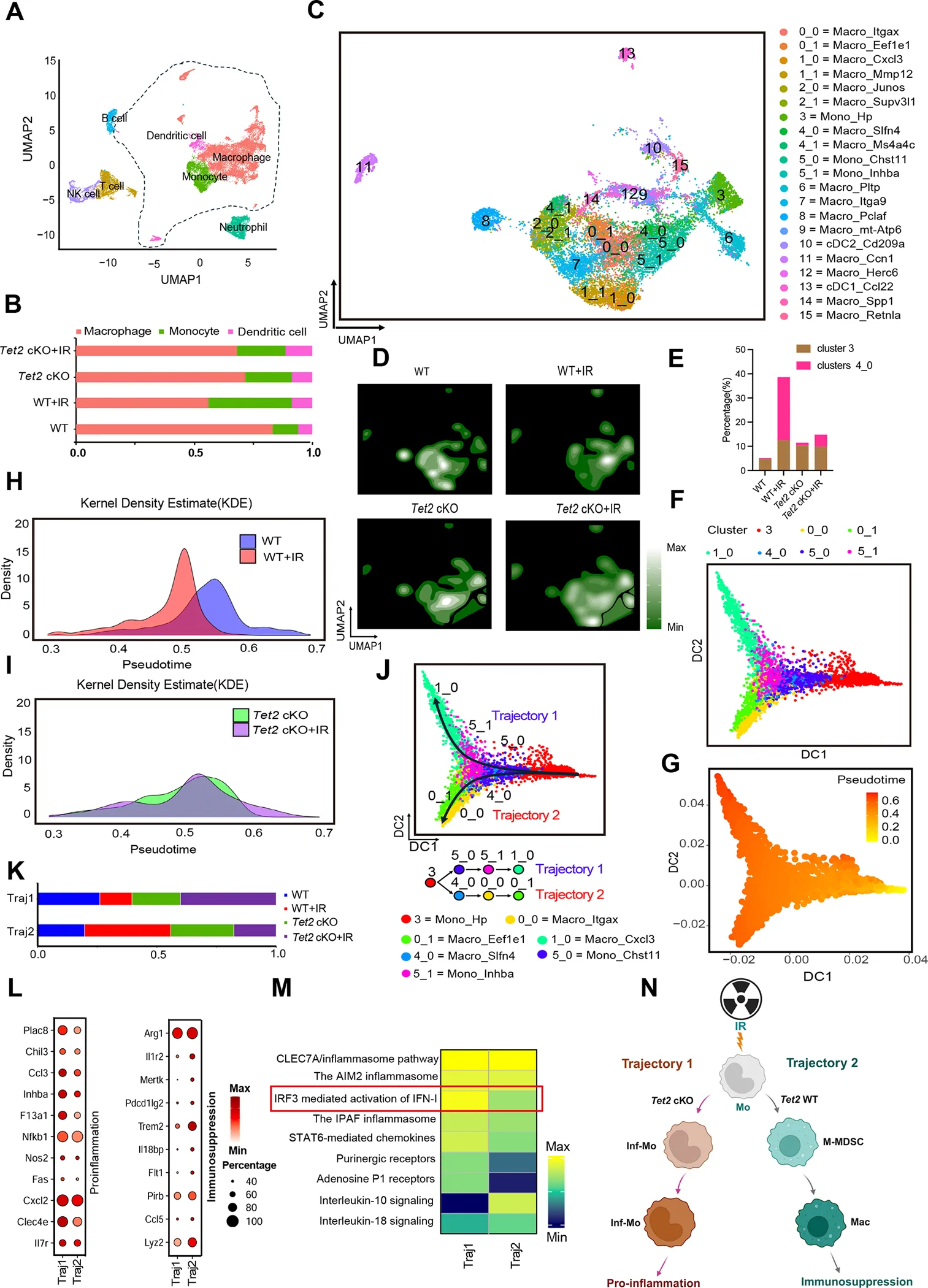
scRNA-seq identifies tumor immune environment changes in irradiated tumors of WT and *Tet2* cKO mice. **(A)** scRNA-seq and unsupervised uniform manifold approximation and projection (UMAP) clustering of live CD45^+^ cells in the MC38 tumor. Outline indicates clusters of myeloid cells. **(B)** Proportions of 3 major groups of myeloid cells stratified by experimental groups as indicated. **(C)** UMAP clustering of myeloid cells from WT and *Tet2* cKO tumors (±20 Gy irradiation), identification of 21 subclusters. **(D)** Density plot of myeloid cells in tumor stratified by experimental groups as indicated. **(E)** Bar plot showing the percentage of cluster 3 and cluster 4_0 cells among total myeloid cells across WT, WT+IR, *Tet2* cKO, and *Tet2* cKO+IR groups. **(F)** scRNA-seq and diffusion map projection of cells in selected 7 clusters in tumors of WT/*Tet2* cKO tumor-bearing mice ± 20 Gy, n = 4 mice per group. DC = diffusion component. **(G)** Pseudotime diffusion map of cells in selected 7 clusters. **(H)** Density plots across pseudotime for cells derived from WT/WT+IR conditions. **(I)** Density plots across pseudotime for cells derived from *Tet2* cKO/*Tet2* cKO+IR conditions. **(J)** Trajectory inference of cells using Slingshot and schematic depiction of two main trajectories. **(K)** Proportion of trajectories 1 and 2 in the four experimental groups. **(L)** Bubble plot showing differential proinflammatory/immunosuppressive genes in trajectories 1 and 2. **(M)** Heatmap of Reactome pathway enrichment of trajectories 1 and 2 correlating to cellular functions. **(N)** Patterns of monocyte differentiation in trajectories 1 and 2.

**Figure 3.**
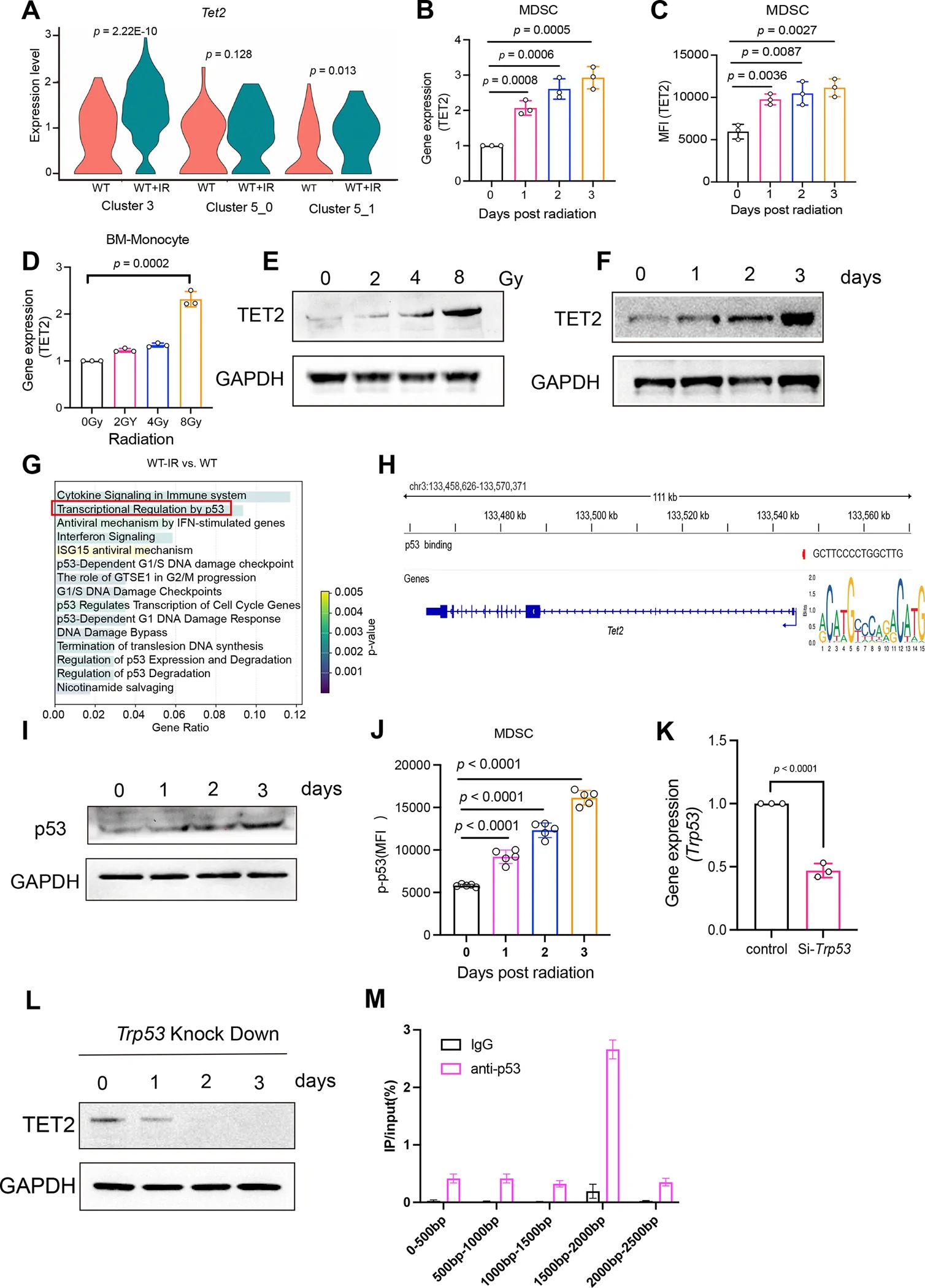
Radiation induces TET2 expression through the p53 pathway. **(A)** Violin plots showing *Tet2* expression distributions across tumor-infiltrating monocyte subclusters (scRNA-seq, CD45^+^ cells, 72 h post-IR). Wilcoxon rank-sum test. **(B)** qPCR analysis of *Tet2* expression in FACS-purified MDSCs from MC38 tumors at days 0–3 after IR. Data are mean ± SD; each dot represents one biological replicate (n = 3). One-way ANOVA with Bonferroni’s multiple comparisons test. **(C)** MFI of TET2 protein in FACS-purified MDSCs from MC38 tumors at days 0–3 after IR. Data are mean ± SD; each dot represents one biological replicate (n = 3). One-way ANOVA with Bonferroni’s multiple comparisons test. **(D)** qPCR analysis of *Tet2* expression in bone marrow-derived MDSCs after 0, 2, 4, or 8 Gy irradiation. Data are mean ± SD; each dot represents one biological replicate (n = 3). One-way ANOVA with Bonferroni’s multiple comparisons test. **(E)** Western blot analysis of TET2 protein expression in bone marrow-derived monocytes at 24 h post-irradiation (0, 2, 4, 8 Gy). **(F)** Western blot analysis of TET2 protein expression kinetics in bone marrow-derived monocytes following 8 Gy irradiation (0–72 h post-exposure). **(G)** Gene ontology analysis of RNA-seq data identified p53-mediated transcriptional regulation as a top enriched pathway. **(H)** Genomic view of the *Tet2* locus showing the predicted p53-binding site on chromosome 3. The corresponding DNA sequence and p53 motif logo are shown on the right. **(I)** Western blot analysis of p53 protein expression kinetics in bone marrow-derived monocytes following 8 Gy irradiation (0–72 h post-exposure). **(J)** MFI of p-p53 in FACS-purified MDSCs from MC38 tumors at days 0–3 after IR. Data are mean ± SD; each dot represents one tumor sample (n = 5 per group). One-way ANOVA with Bonferroni’s multiple comparisons test. **(K)** qPCR validation of *Trp53* knockdown. Data are mean ± SD; n = 3 replicates per group. Unpaired two-tailed Student’s t-test. **(L)** Western blot analysis of TET2 kinetics in *Trp53*-knockdown bone marrow-derived monocytes after 8 Gy irradiation (0–72 h post-exposure). **(M)** ChIP-qPCR analysis of p53 enrichment at the indicated *Tet2* promoter regions in bone marrow-derived monocytes. Data are mean ± SD; n = 3 biological replicates.

**Figure 4.**
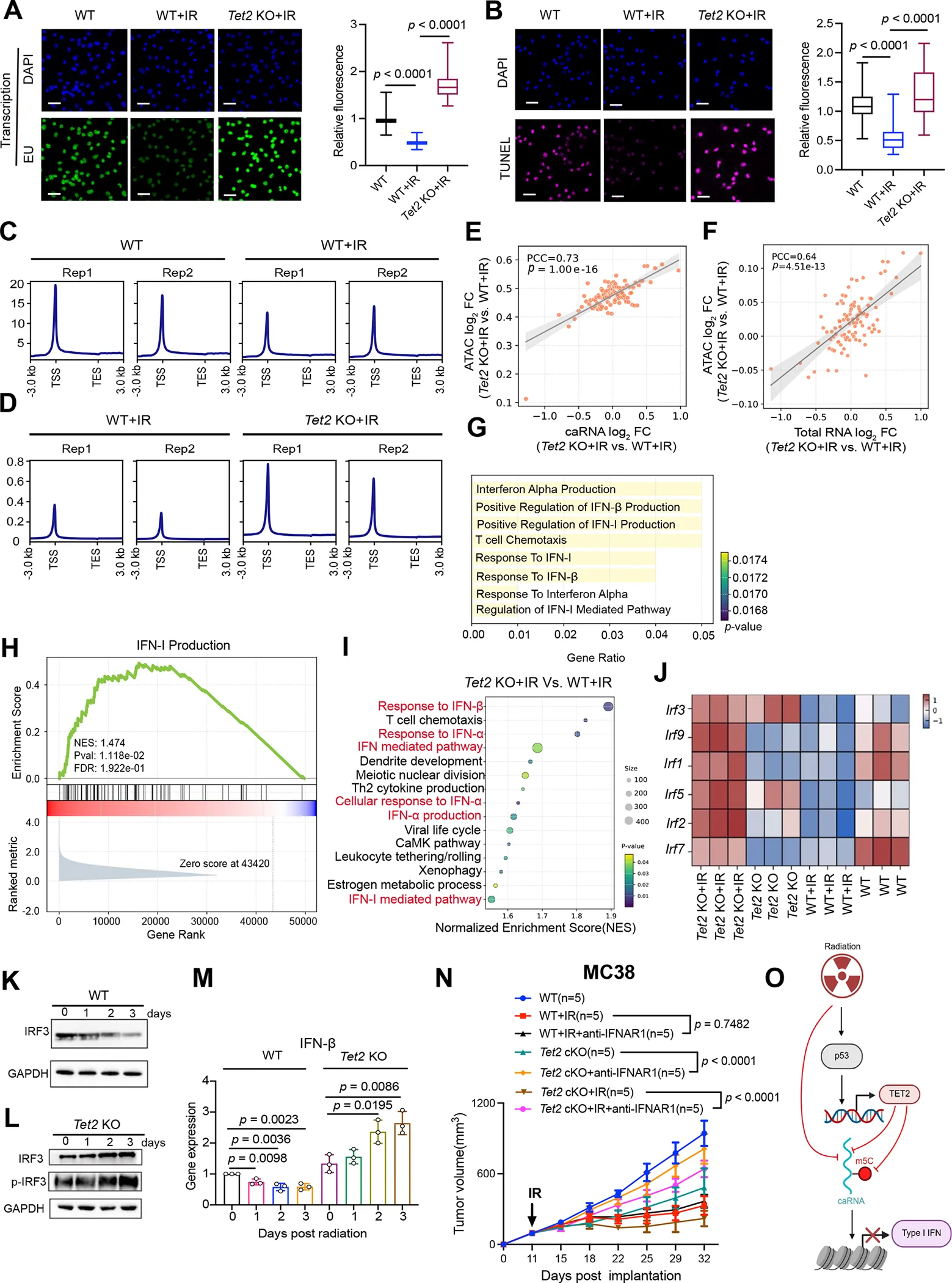
A Radiation-Specific caRNA m^5^C Epigenetic Switch Licenses Myeloid-Driven Immune Evasion in Solid Tumors. **(A)** EU incorporation assay of global nascent RNA synthesis; DAPI stains nuclei. Right, quantification of EU intensity. Box plots show median, interquartile range, and min–max whiskers. Scale bars, 10 μm. One-way ANOVA with Bonferroni’s test. **(B)** DNase-TUNEL assay of global chromatin accessibility; DAPI stains nuclei. Right, quantification of TUNEL intensity. Box plots show median, interquartile range, and min–max whiskers. Scale bars, 10 μm. One-way ANOVA with Bonferroni’s test. **(C and D)** ATAC-seq peak signals were compared between experimental groups (C: WT vs WT+IR; D: WT+IR vs *Tet2* KO+IR). **(E)** Genome-wide pearson correlation between chromatin accessibility and caRNA expression in the context of IR(Pearson correlation coefficient = 0.73, *p* = 1.00 × 10^−16^). **(F)** Genome-wide pearson correlation between chromatin accessibility and gene expression in the context of IR(Pearson correlation coefficient = 0.64, *p* = 4.51 × 10^−13^). **(G)** GO enrichment analysis of RNA-seq DEGs in *Tet2* KO+IR versus WT+IR monocytes. Bars indicate gene ratio; color indicates enrichment p value. **(H)** GSEA of the type I interferon production gene set using ATAC-seq-ranked genes. Running enrichment score, gene set hits, NES, nominal p value, and FDR are shown. **(I)** GO bubble plot showing enriched pathways in *Tet2* KO+IR versus WT+IR monocytes. Pathways with FDR < 0.05 are shown. **(J)** Heatmap of IFN-I mediator expression (*Irf1, Irf2, Irf3, Irf5, Irf7,* and *Irf9*) across the indicated groups. Red indicates higher and blue lower row-scaled normalized expression. **(K)** Immunoblot analysis of IRF3 expression in bone marrow derived monocytes(WT) post-radiation (day 0–3). **(L)** Immunoblot analysis of IRF3/pIRF3expression in bone marrow derived monocytes (*Tet2* KO) post-radiation (day 0–3). **(M)** qPCR analysis of IFN-β expression in MC38 tumor-infiltrating MDSCs from WT and *Tet2* KO mice after IR. Data are mean ± SD; n = 3 replicates per group. One-way ANOVA with Bonferroni’s multiple comparisons test. **(N)** MC38 tumor-bearing WT and *Tet2* cKO mice received local IR (20 Gy) when tumors reached approximately 100 mm^3^, followed by anti-IFNAR1 mAb treatment (200 μg, i.t., twice weekly) starting on day 0. Tumor growth was monitored. Data are shown as mean ± SD (n = 5 mice per group). Two-way ANOVA with correction for multiple comparisons. **(O)** The Scheme. Radiation induces p53-mediated TET2 upregulation, reducing caRNA expression and oxidizing m^5^C to promote chromatin compaction and suppress type I interferon responses.

**Figure 5.**
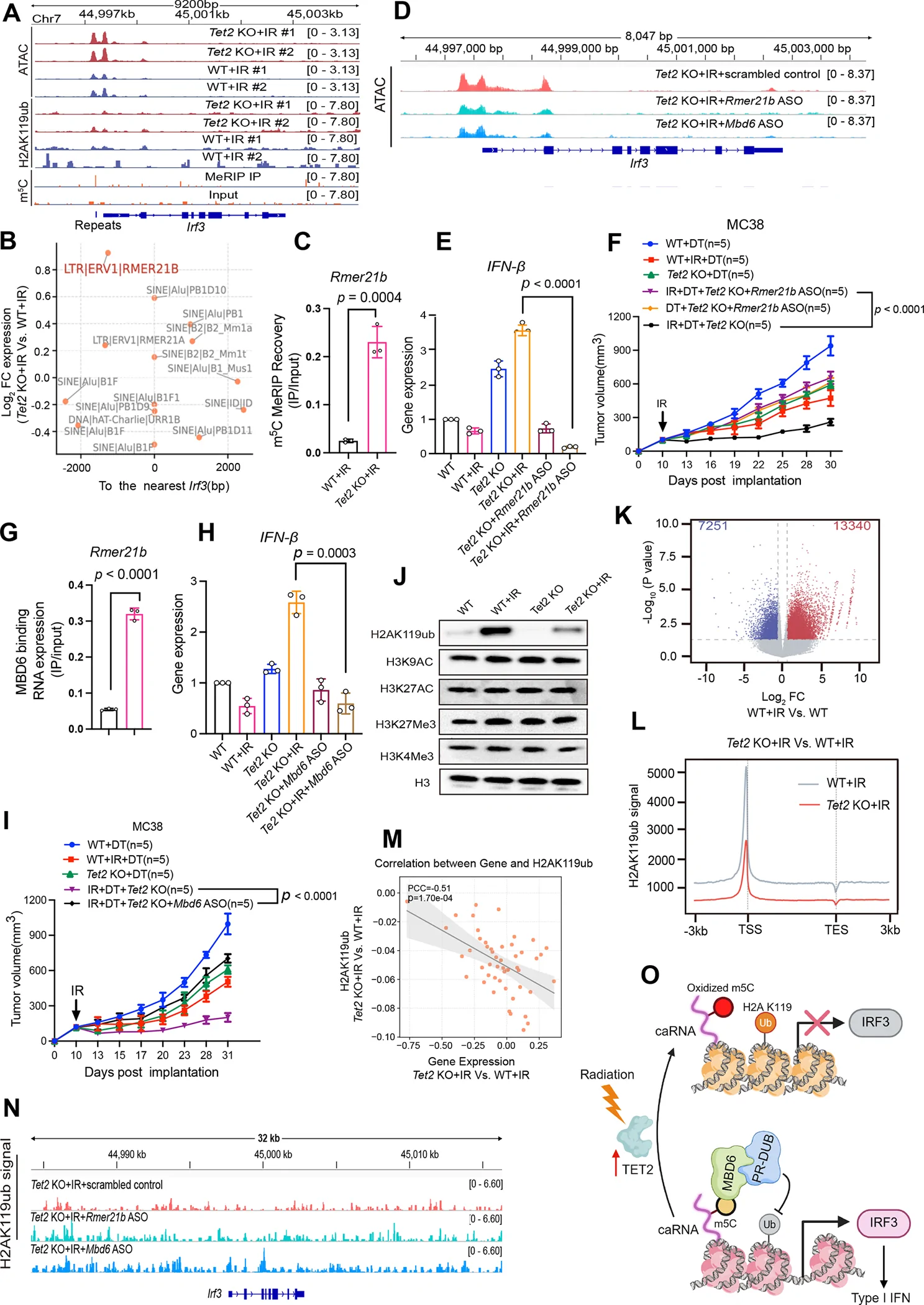
The caRNA-MBD6-H2AK119ub axis activates IRF3-mediated interferon signaling upon irradiation. **(A)** Genome browser tracks of ATAC-seq, H2AK119ub, and m^5^C MeRIP signals at the *Irf3* promoter across conditions **(B)** Differential chromatin-associated RNA (caRNA) expression by caRNA-seq at the *Irf3* promoter region in monocytes of *Tet2* KO+IR compared to WT+IR. **(C)** MeRIP-qPCR analysis of m^5^C methylation on *Rmer21b* in *Tet2* KO+IR versus WT+IR monocytes, normalized to input. Data are mean ± SD; n = 3 replicates per group. Unpaired two-sided t-test. **(D)** ATAC-seq tracks at the *Irf3* promoter under indicated conditions (scale bar: 8 kb). **(E)** *Ifnb* mRNA expression by qPCR (normalized to *Gapdh*). Data are shown as mean ± SD; each dot represents one replicate (n = 3 per group). One-way ANOVA with Bonferroni’s test. **(F)** Tumor bearing *Lyz*^Cre^CSF1R-DTR mice received DT, followed by tumor-localized IR and transfer of WT, *Tet2* KO, or *Tet2* KO+*Rmer21b*-ASO–treated monocytes. Tumor growth was monitored. Data are mean ± SD; n = 5 mice per group. Two-way ANOVA with multiple-comparison correction **(G)** MBD6 CLIP-PCR analysis of m^5^C-modified *Rmer21b* transcripts in *Tet2* KO+IR versus WT+IR monocytes. Data are mean ± SD; n = 3 replicates per group. Unpaired two-sided t-test. **(H)** qPCR analysis of *Ifnb1* mRNA expression normalized to *Gapdh*. Data are mean ± SD; n = 3 replicates per group. One-way ANOVA with Bonferroni’s test. **(I)** Tumor bearing *Lyz*^Cre^CSF1R-DTR mice received DT, followed by tumor-localized IR and transfer of WT, *Tet2* KO, or *Tet2* KO+*Mbd6*-ASO–treated monocytes. Tumor growth was monitored. Data are mean ± SD; n = 5 mice per group. Two-way ANOVA with multiple-comparison correction. **(J)** Western blot analysis of histone modification profiles across four experimental groups (WT, WT+IR, *Tet2* KO, *Tet2* KO+IR), probing for H2AK119ub, H3K9AC, H3K27AC, H3K27Me3, H3KMe3. **(K)** Volcano plot displaying differential H2AK119ub deposition identified by Cut&Tag in WT+IR versus WT conditions. **(L)** The H2AK119ub Cut&Tag peak signal across genomic regions in *Tet 2*KO+IR (red) and WT+IR (blue) samples. **(M)** Scatter plot analysis displays an inverse relationship between H2AK119ub signal intensity and corresponding gene expression levels (pearson correlation coefficient = −0.59, *p* = 1.07 × 10^−10^). **(N)** H2AK119ub peaks at the *Irf3* gene region under three experimental conditions: *Tet2* KO+IR (red), *Tet2* KO+IR with *Rmer21b*-targeting ASO (green), and *Tet2* KO+IR with *Mbd6*-targeting ASO (blue) (scale bar: 32 kb). **(O)** Schematic diagram. Radiation induces p53-mediated TET2 upregulation, which oxidizes m^5^C in chromatin-associated RNAs, promoting chromatin compaction and suppressing type I interferon responses.

**Figure 6.**
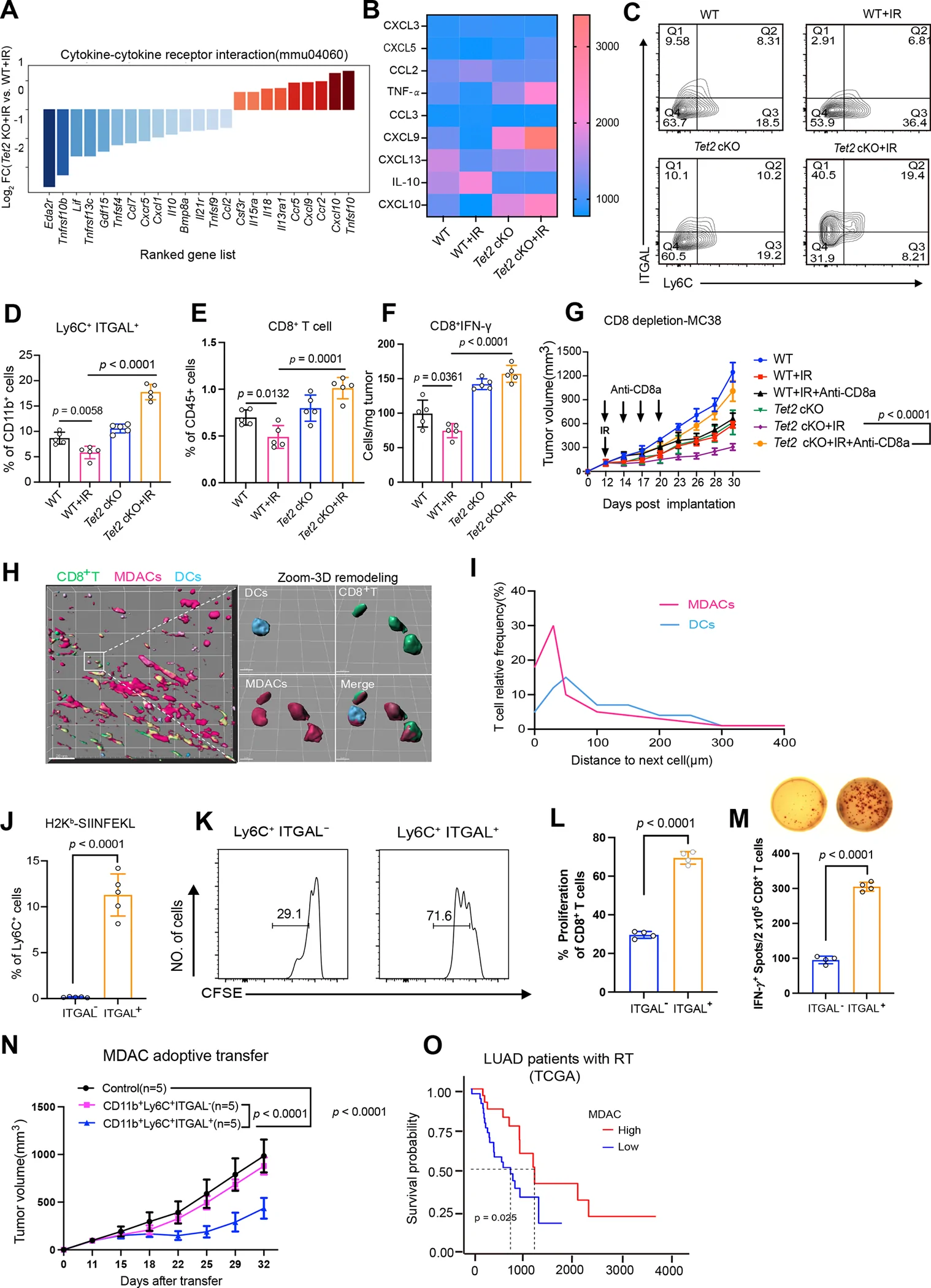
Inflammatory monocytes stimulate T cells within the TME. **(A)** RNA-seq identified 23 differentially expressed proinflammatory cytokine genes in *Tet2* cKO+IR versus WT+IR (FDR < 0.05, |log2FC| > 1, DESeq2 with Benjamini-Hochberg correction). **(B)** Cytokine levels in MC38 tumors 72h post-IR were quantified using LEGENDplex (13-plex) and normalized to tumor mass (pg/mg; n=5/group). **(C and D)** Frequency of proinflammatory MDACs (CD11b^+^Ly6C^+^ITGAL^+^) (C) across the indicated conditions (D). Data are shown as mean ± SD; each dot represents one tumor (n = 5 per group). One-way ANOVA with Bonferroni’s multiple comparisons test. **(E and F)** Flow cytometric analysis of tumor-infiltrating CD8^+^ T cells (E) and CD8^+^ IFN-γ^+^ T cells (F) in WT or *Tet2* cKO mice 3 days after IR. Data are mean ± SD; each dot represents one tumor (n = 5 per group). One-way ANOVA with Bonferroni’s multiple comparisons test. **(G)** WT and *Tet2* cKO mice bearing MC38 tumors (100 mm^3^) received tumor-localized IR (20 Gy) with concurrent αCD8 depletion (200μg/mouse twice weekly). Tumor growth was monitored. Data are shown as mean ± SD (n = 6 mice per group).Two-way ANOVA test with corrections for multiple variables. **(H)** Multiplex immunofluorescence of MDACs (ITGAL^+^), DCs (FSCN1^+^), and CD8^+^ T cells in MC38 tumors from *Tet2* cKO+IR mice (left), with zoomed fields (right) showing cellular interactions. Representative 5-μm z-stack projections from three independent experiments (scale bars: 100 μm, overview; 20 μm, zoom). **(I)** Relative T cell frequency and distance to the next immune cell (MDACs in red and DCs in blue) in *Tet2* cKO+IR group. **(J)** Flow cytometric quantification of Ly6C^+^H-2Kb-SIINFEKL^+^ cell frequencies in ITGAL^−^ versus ITGAL^+^ populations. Data are shown as mean ± SD; each dot represents one tumor (n = 5 per group). Unpaired two-tailed Student’s t-test. **(K and L)** CFSE dilution assay of T cells co-cultured with Ly6C^+^ITGAL^+^ or Ly6C^+^ITGAL⁻ monocytes isolated from *Tet2* cKO+IR tumors (K). Histograms show CFSE distributions (L). Data are shown as mean ± SD; each dot represents one tumor (n = 4 per group).Unpaired two-tailed Student’s t-test. **(M)** ELISPOT assay of IFN-γ^+^ spots from CD8^+^ T cells co-cultured with Ly6C^+^ITGAL^+^ or Ly6C^+^ITGAL⁻ monocytes isolated from *Tet2* cKO+IR tumors. Data are mean ± SD; n = 4 replicates per group. Unpaired two-tailed Student’s t-test. **(N)** WT mice bearing MC38 tumors were treated with MDAC adoptive transfer, tumor growth was monitored. Data are shown as mean ± SD (n = 5 mice per group). two-way ANOVA with correction for multiple comparisons. **(O)** Kaplan-Meier survival curves for high vs. Low MDAC signature in radiotherapy-treated lung cancer cohorts (TCGA, median cutoff). Log-rank test.

**Figure 7.**
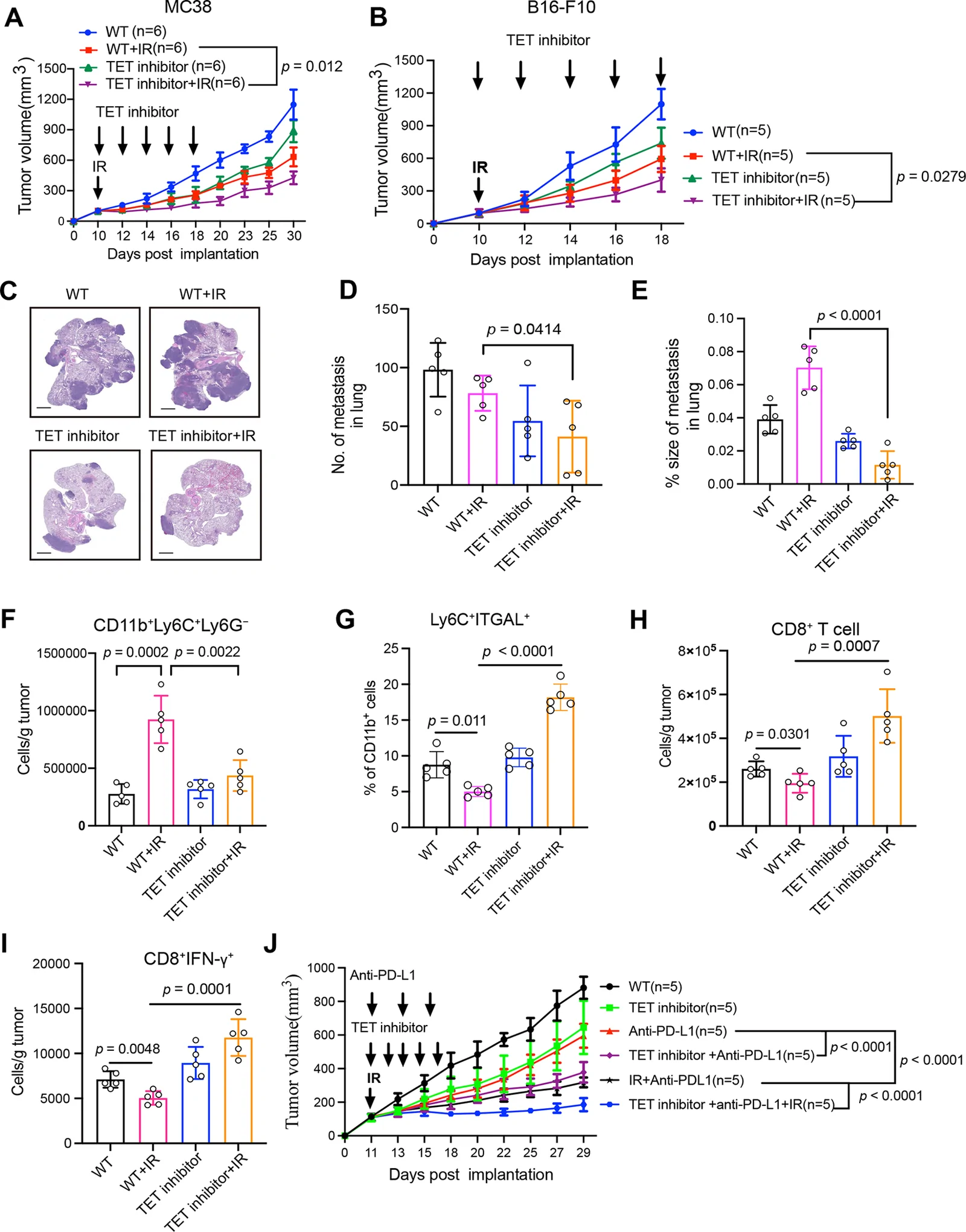
Pharmacological targeting of TET2 potentiates therapeutic responses to radiotherapy and immunotherapy. **(A and B)** Tumor growth in MC38 (A) and B16-F10 (B) tumor-bearing WT mice treated with local IR (20 Gy) and TET inhibitor (10 μM, q.o.d. ×5). Data are shown as mean ± SD; n = 6 mice per group in (A) and n = 5 mice per group in (B). Two-way ANOVA. **(C-E)** H&E-stained lung sections (C). Scale bars, 2 mm. Quantification of metastasis number (D) and mean size of five largest lesions per lung area (E). Data are shown as mean ± SD; each dot represents one lung(n = 5/group). One-way ANOVA with Bonferroni’s test. **(F-G)** Tumor-infiltrating CD11b^+^Ly6C^+^Ly6G⁻ (F) and CD11b^+^Ly6C^+^ITGAL^+^ (G) cells in MC38 tumor-bearing mice. Data are shown as mean ± SD; each dot represents one tumor(n = 5/group).One-way ANOVA with Bonferroni’s test. **(H-I)** Quantification of tumor-infiltrating CD8^+^ T cells (H) and CD8^+^IFNγ^+^ T cells (I) in MC38 tumor-bearing mice under indicated treatments. Data are shown as mean ± SD; each dot represents one tumor(n = 5/group).One-way ANOVA with Bonferroni’s test. **(J)** MC38 tumor-bearing WT mice received indicated treatments: local IR (20 Gy), anti-PD-L1 (200 μg, biw ×3), and/or TET inhibitor (10 μM, q.o.d.). Tumor growth monitored. Data are shown as mean ± SD (n = 5 mice per group). Two-way ANOVA with multiple comparison correction.

**Key Resources Table:** 

Reagent or Resource	Source	Identifier

Antibodies		

Anti-mouse CD45	Biolegend	Cat#: 103126; RRID: AB_493535
Anti-mouse CD11b	Biolegend	Cat#: 101222; RRID: AB_493705
Anti-mouse CD11c	Biolegend	Cat#: 117318; RRID: AB_493568
Anti-mouse F4/80	Biolegend	Cat#: 111603; RRID: AB_3082990
Anti-mouse Ly6C	Biolegend	Cat#: 128005; RRID: AB_1186134
Anti-mouse Ly6G	Biolegend	Cat#: 127639; RRID: AB_2565880
Anti-mouse CD3	Biolegend	Cat#: 100219; RRID: AB_1732068
Anti-mouse CD4	Biolegend	Cat#: 100411; RRID: AB_312696
Anti-mouse CD8a	Biolegend	Cat#: 100706; RRID: AB_312745
Anti-mouse Gr1+	Biolegend	Cat#: 108405; RRID: AB_313370
Anti-mouse IFNγ	Biolegend	Cat#: 505826; RRID: AB_2295770
Anti-human FoxP3 Anti-human CD20 Anti-human CD16 Anti-human CD4 Anti-human CD34 Anti-human CD11c Anti-human CD303 Anti-human CD304 Anti-human CD56 Anti-human CD45 Anti-human CD33 Anti-human CD8a Anti-mouse CD8a Anti-mouse FSCN1 Anti-mouse ITGAL	Invitrogen Invitrogen Invitrogen Biolegend Biolegend Biolegend Biolegend Biolegend Biolegend ThermoFisher ThermoFisher ThermoFisher Biolegend Santa Cruz Biolegend	Cat#:41-4777-82; RRID:AB_2573609 Cat#:11-0209-42; RRID:AB_2572440 Cat#:15-0168-42; RRID:AB_2573060 Cat#:980812; RRID:AB_2820224 Cat#:343613; RRID:AB_2571926 Cat#:301625; RRID:AB_10662901 Cat#:314403; RRID:AB_2904342 Cat#:354509; RRID: AB_2561558 Cat#:354509; RRID: AB_2561558 Cat#:MHCD4501; RRID: AB_10371768 Cat#:45-0338-42; RRID:AB_10714975 Cat#:25-0087-42; RRID:AB_10733019 Cat#:100703; RRID: AB_312742 Cat#:sc-21743; RRID:AB_627580. Cat#:141006;RRID:AB_10694371
Anti-TET2	Abcam	Cat#: ab94580; RRID: AB_10887588
Anti-mouse p53	Cell Signaling Technology	Cat#: 2524S; RRID: AB_3697874
Anti-5-methylcytosine (5-mC)	Diagenode	Cat#: C15200081-100; RRID: AB_2572207
β-Actin antibody GAPDH antibody	Santa Cruz Cell Signaling Technology	Cat#: sc-47778; RRID: AB_626632 Cat#: 2118S; RRID: AB_3697875
In vivo CD8α antibody	Bio X Cell	Cat#: BE0004-1; RRID: AB_1107671
Fc Block InVivoMAb anti-mouse PD-L1 (B7-H1) InVivoMAb Anti-Mouse IFNAR-1	Bio X Cell Bio X Cell Leinco Technology	Cat#: BE0307; RRID: AB_2736987 Cat#: BE0361; RRID: AB_2927503 Cat#: I-401; RRID: AB_2491621

Chemicals, peptides, or recombinant proteins		

Collagenase, Type I	Fisher Scientific	Cat#: LS004197
DNase I	Sigma	Cat#: 10104159001
Cell Activation Cocktail	Biolegend	Cat#: 423303
Actinomycin D	Sigma	Cat#: A9415-5MG
UltraComp Compensation Beads	Fisher	Cat#: 01-2222-42
Protease inhibitor	Thermo Fisher	Cat#: 1861278
GeneSilencer siRNA Transfection Reagent	Amsbio	Cat#: AMS.T500020
Zombie NIR^™^	Biolegend	Cat#: 423106
Proteinase K Solution	Invitrogen	Cat#: 25530049
Dynabeads^™^ Protein G for Immunoprecipitation	Invitrogen	Cat#: 10003D

Critical commercial assays		

Mouse IFN-γ ELISPOT set	Fisher Scientific	Cat#: BDB551873
Magna RIP^™^ RNA-Binding Protein Immunoprecipitation Kit	Sigma	Cat#: 17-700
SMARTer^®^ Stranded Total RNA-Seq Kit v2 - Pico Input Mammalian	Takara	Cat#: 634413
Dynabeads^™^ MyOne^™^ Streptavidin C1	Fisher	Cat#: 65001
EasySep^™^ Mouse CD8^+^ T cell Isolation Kit EasySep Mouse Monocyte Isolation Kit STEMCELL LEGENDplex Mouse Inflammation Panel (13-plex)	Stemcell Stemcell BioLegend	Cat#: 19853 Cat# 19861 Cat# 740446
Power SYBR Green PCR Master Mix Fixation and Permeabilization Solution CFSE Cell Proliferation Kit	Thermo Fisher BD Bioscience Invitrogen	Cat#: 4367659 Cat# 554722 Cat# C34554
High Capacity cDNA reverse transcription Kit	Thermo Fisher	Cat#: 4374966
NEBNext^®^ Poly(A) mRNA Magnetic Isolation Module	NEB	Cat#: E7490L
Magna CHIP^™^ A/G Chromatin Immunoprecipitation Kit	Sigma	Cat#: 17-10085
RNA Clean & Concentrator-5	Zymo Research	Cat#: R1013
Direct-zol RNA Microprep	Zymo Research	Cat#: R2061

Deposited Data		

ATAC, mRNA-seq, meRIP-seq, Cut & tag, caRNA-seq	This paper	PRJNA1305028
Single cell seq	This paper	GSE304925
TCGA dataset	The Cancer Genome Atlas	https://portal.gdc.cancer.gov/

Experimental Model: Cell lines		

MC38	ATCC	CRL-2640
B16F10-OZ	Maintained in our lab	N/A
LLC	ATCC	CRL-1642

Experimental Model: Organisms/Strains		

C57BL/6 mice	Harlan Envigo	N/A
Tet2^f/f^ mice	JAX	N/A
*Lyz* ^Cre^ *Tet2* ^fl/fl^	JAX	N/A

Oligonucleotides		

LTR-RMER21B ASO	AUM BioTech	N/A
MBD6 ASO CXXC5 ASO	AUM BioTech AUM BioTech	N/A N/A

Biological samples		

Patient PBMCs	University of Chicago	NCT03223155

Software and algorithms		

GraphPad Prism	GraphPad	https://www.graphpad.com/scientific-software/prism/
FlowJo (10.10.0)	FlowJo	https://www.flowjo.com/solutions/flowjo
Seurat package (v4.0.6)	Stuart et al.^62^	https://github.com/satijalab/seurat/releases/tag/v4.0.6
scClassify (v1.2.0)	Lin et al.^63^	https://github.com/SydneyBioX/scClassify_analysis
Trimmomatic-0.39	Bolger, et al.^64^	http://www.usadellab.org/cms/index.php?page=trimmomatic
HISAT (version 2.1.0)	Kim et al.^65^	https://github.com/DaehwanKimLab/hisat2
MACS (version 2)	Zhang et al.^66^	http://liulab.dfci.harvard.edu/MACS/
samtools (version 1.9) bedtools (v.2.26.0) HTSeq	Danecek et al.^67^ Danecek et al.^67^ Anders et al.^68^	http://www.htslib.org/ http://www.htslib.org/ http://www-huber.embl.de/HTSeq
