# Supplementary material for: Myeloid reprogramming generates myeloid-derived activating cells to potentiate radiotherapy and suppress metastasis
Source: Cancer Cell. Author manuscript; Available in PMC 2026 Oct 15. (PMC13669204; doi:10.1016/j.ccell.2026.08.017)
Supplement: Supplementary file 1 [file NIHMS2212433-supplement-Supplementary_final0812.pdf]

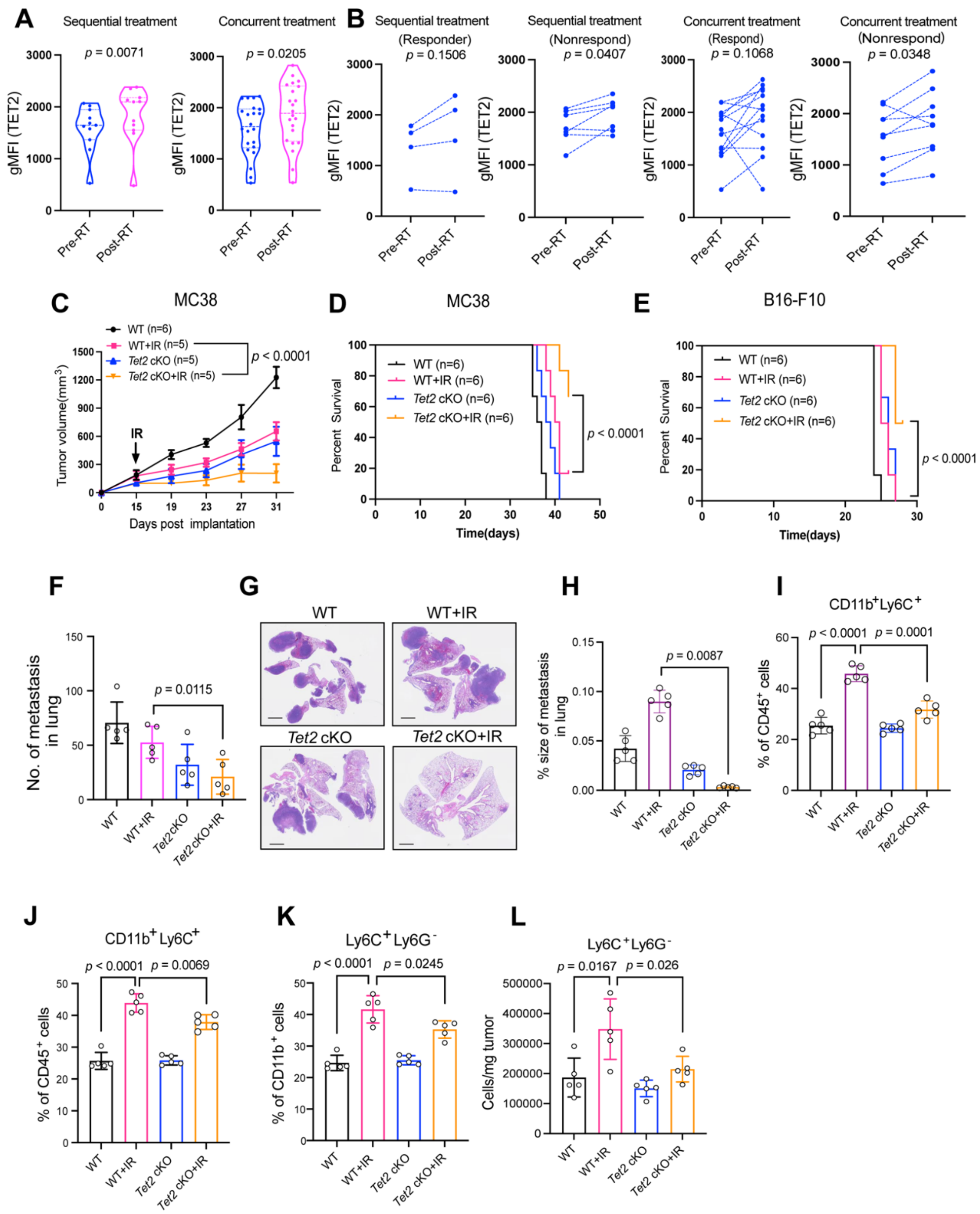

Figure S1. T*et2* deficiency in myeloid cells improves response to radiotherapy

(A) Geometric mean fluorescence intensity (gMFI) of TET2 from M-MDSCs pre- and post-RT in sequential treatment patients, concurrent treatment patients. the center dashed line indicates the median, and the dotted lines indicate the interquartile range. Paired two-sided t-test.

(B) gMFI of TET2 from M-MDSCs pre- and post-RT in responders/nonresponders of sequential and concurrent treatment patients. Paired two-sided t-test.

(C) Wild-type (*Tet2*$^{fl/fl}$) or *Tet2* cKO (*Lyz*^{Cre}*Tet2*$^{fl/fl}$) mice were injected subcutaneously with 1x10^{6}

MC38 cells. When the tumor size reached 100 mm^3, tumor-bearing mice were treated with tumor-local IR (20 Gy, one dose). Tumor growth was monitored. Data are shown as mean ± SD; n = 6 for WT and n = 5 for WT+IR, *Tet2* cKO, and *Tet2* cKO+IR. Two-way ANOVA test with corrections for multiple variables.

(D and E) Wild-type (*Tet2*$^{fl/fl}$) or *Tet2* cKO (*Lyz*^{Cre}*Tet2*$^{fl/fl}$) mice were injected subcutaneously with 1 or 2x10^6 MC38 (D) or B16-F10 (E) cells. When the tumor size reached 100 mm^3, tumor-bearing mice were treated with tumor-local IR (20 Gy, one dose). Survival was monitored. n = 6 mice per group. Two-sided log rank (Mantel-Cox) test.

(F) Number of lung metastases (LLC tumor) in WT or *Tet2* cKO mice 30 days after tumor implantation. Data are shown as mean ± SD; each dot represents one lung (n = 5 per group). One-way ANOVA with Bonferroni's multiple comparison tests.

(G) Representative H&E stainings of lungs of flank LLC-tumor-bearing mice 30 days after tumor implantation. Primary tumors were surgically resected 7 days post-irradiation in all four experimental groups, with treatments as indicated. Scale bars, 2 mm.

(H) Mean size of the five largest LLC lung metastases per lung area 30 days post-implantation (n = 5 per group). Primary tumors were resected 7 days after irradiation. Data are shown as mean ± SD; each dot represents one lung (n = 5 per group). One-way ANOVA with Bonferroni's multiple comparison test.

(I) The percentage of tumor-infiltrating $CD11b^+Ly6C^+$ cells in LLC lung metastases 30 days post-implantation, assessed by flow cytometry. Primary tumors were resected 7 days after irradiation. Data are shown as mean ± SD; each dot represents one tumor (n = 5 per group). One-way ANOVA with Bonferroni's multiple comparison test.

(J) The percentage of tumor-infiltrating $CD11b^+Ly6C^+$ cells in WT or *Tet2* cKO mice three days after IR, as assessed by flow cytometry. Data are shown as mean ± SD; each dot represents one tumor (n = 5 per group). One-way ANOVA with Bonferroni's multiple comparison tests.

(K and L) The percentage(K) and cell number(L) of tumor-infiltrating $Ly6C^+Ly6G^-$ cells in WT or *Tet2* cKO mice three days after IR, as assessed by flow cytometry. Data are shown as mean ± SD; each dot represents one tumor (n = 5 per group). One-way ANOVA with Bonferroni's multiple comparison tests.

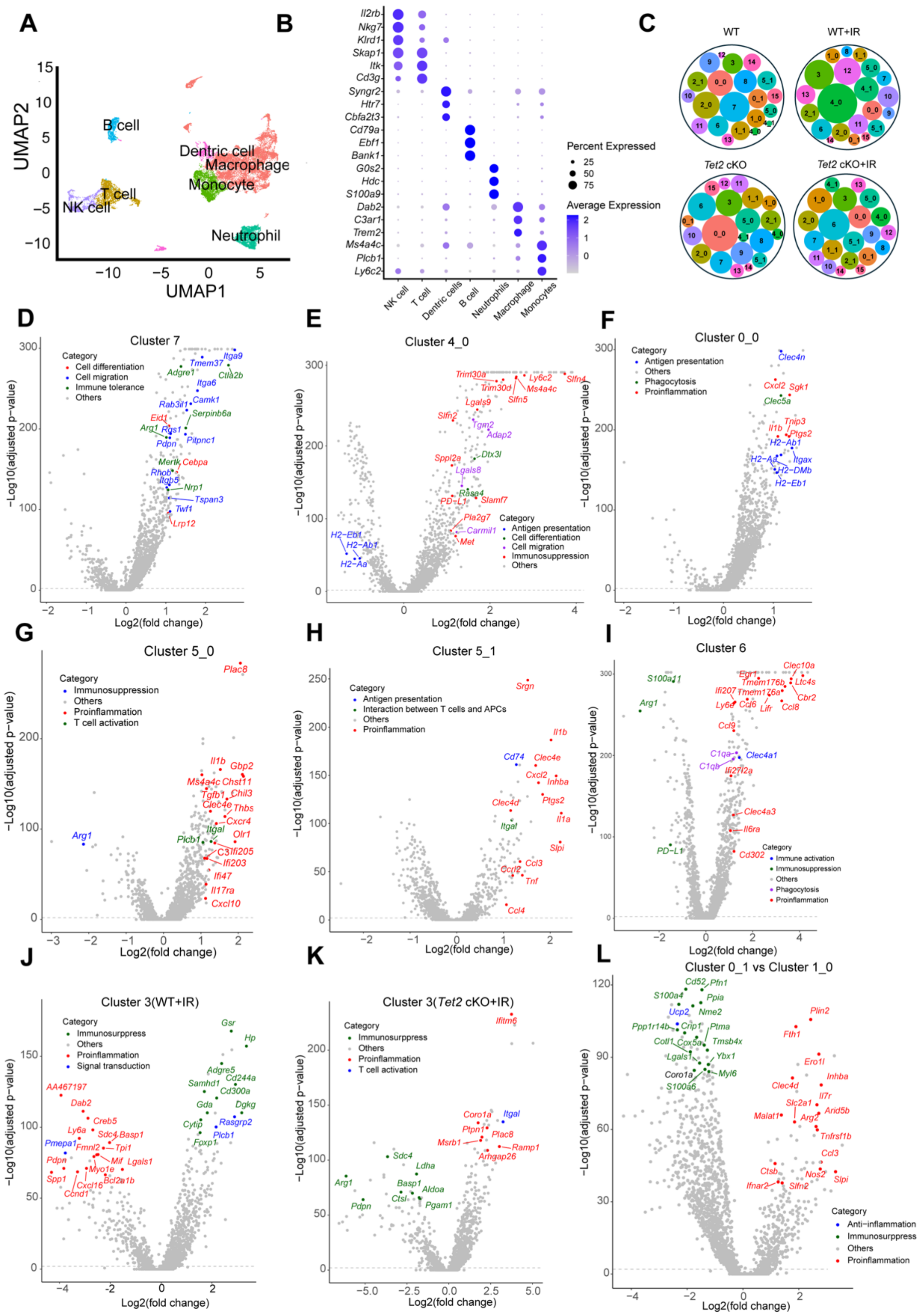

Figure S2. scRNA-seq identifies main clusters and marker genes in the four experimental groups.

(A) scRNA-seq profiling of live CD45$^+$ cells from WT and *Tet2* cKO tumors (±20 Gy irradiation) identified 7 major immune lineages by UMAP clustering (n=4 mice/group).

(B) Bubble plot showing expression of top three marker genes for 7 major cell lineages. DEGs determined by Wilcoxon rank-sum test.

(C) Dot plot representing the number of cells of the 21 myeloid subclusters stratified by 4 experimental groups.

(D) Volcano plot of up- and downregulated genes in cluster 7 (Macro_Itga9). Genes are colored by function. Wilcoxon rank-sum test.

(E) Volcano plot of up- and downregulated genes in cluster 4_0 (Macro_Slfn4). Genes are colored by function. Wilcoxon rank-sum test.

(F) Volcano plot of up- and downregulated genes in cluster 0_0 (Macro_Itgax). Genes are colored by function. Wilcoxon rank-sum test.

(G) Volcano plot of up- and downregulated genes in cluster 5_0 (Mono_Chst11). Genes are colored by function. Wilcoxon rank-sum test.

(H) Volcano plot of up- and downregulated genes in cluster 5_1 (Mono_Inhba). Genes are colored by function. Wilcoxon rank-sum test.

(I) Volcano plot of up- and downregulated genes in cluster 6 (Macro_Pltp). Genes are colored by function. Wilcoxon rank-sum test.

(J) Volcano plot of up- and downregulated genes in cluster 3 (Macro_Hp) of WT+IR group. Genes are colored by function. Wilcoxon rank-sum test.

(K) Volcano plot of up- and downregulated genes in cluster 3 (Macro_Hp) of *Tet2* cKO+IR group. Genes are colored by function. Wilcoxon rank-sum test.

(L) Volcano plot of up- and downregulated genes in cluster 0_1(Macro_Eef1e1) and 1_0 (Macro_Cxcl3) group. Genes are colored by function. Wilcoxon rank-sum test.

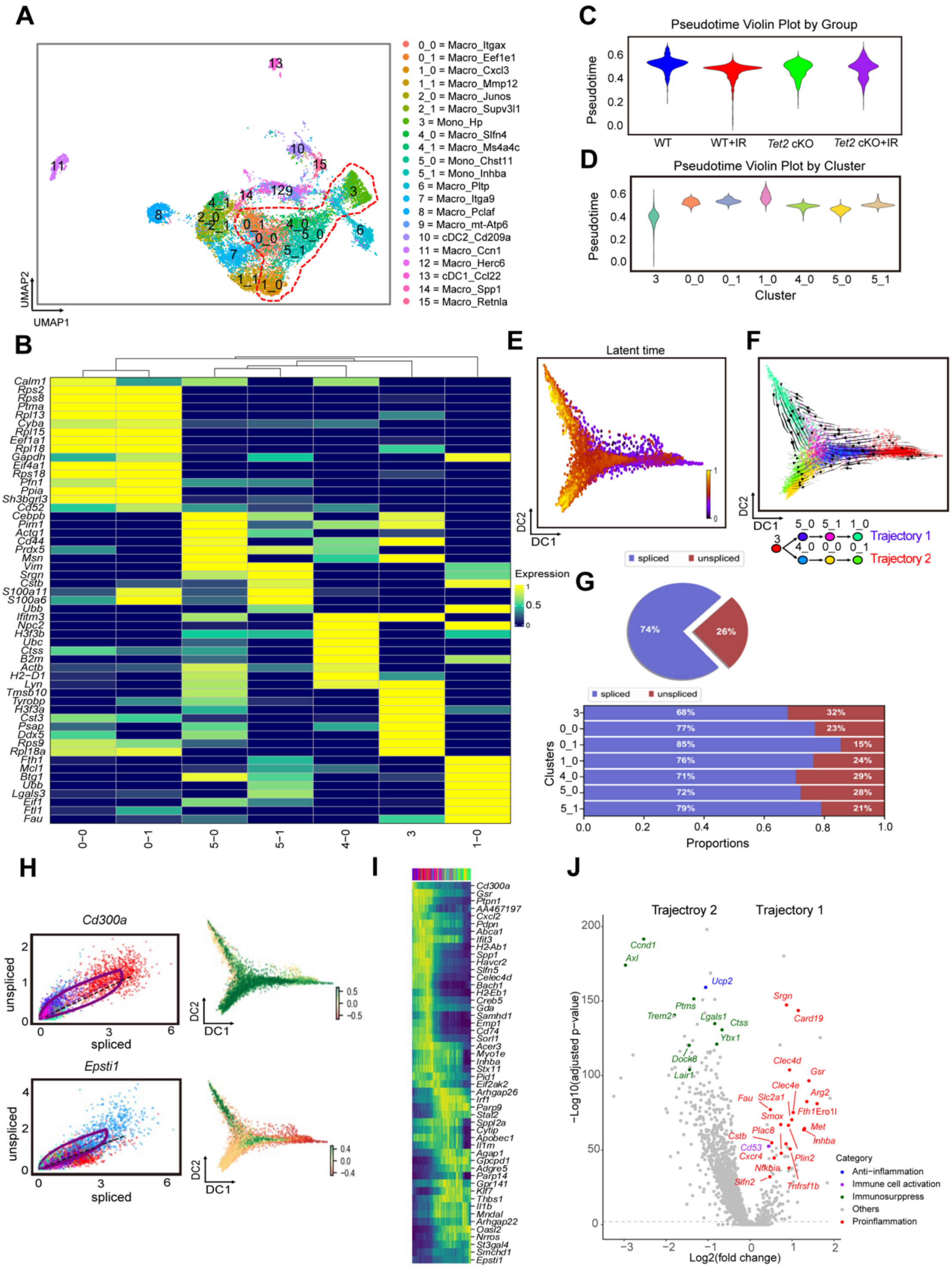

Figure S3. Pseudotime and trajectory analysis of myeloid cells identified by scRNA-seq.

(A) UMAP clustering of myeloid cells identified by scRNA-seq in the tumors of WT and *Tet2* cKO tumor-bearing mice ± 20 Gy, n = 4 mice per group. Clusters used for pseudotime analysis outlined.

(B) Heatmap of marker genes of the 7 clusters used for trajectory analysis.

(C) Violin plot of pseudotime values for 4 groups, categorized by experimental condition.

(D) Violin plot of pseudotime values for 7 populations used for trajectory analysis.

(E) Latent time. The dynamical model recovers the latent time of the underlying cellular processes.

(F) RNA velocity vectors of cells on diffusion map.

(G) Pie chart of total cell clusters RNA splicing ratios detected by scVelo (upper). Bar plot of splicing ratios across seven clusters (down).

(H) Phase portraits of unspliced vs. spliced RNA ratios for *Cd300a* and *Epsti1*. Right: inferred velocity projected onto diffusion map.

(I) Time-series heatmap of the top 50 velocity-informed driver genes across the inferred myeloid trajectory. The top color annotation indicates cell/state ordering. Yellow-green indicates higher and blue-purple lower scaled relative expression.

(J) Volcano plot of significantly enriched genes identified by differential expression analysis in trajectories 1 and 2. Genes are colored by function. Wilcoxon rank-sum test.

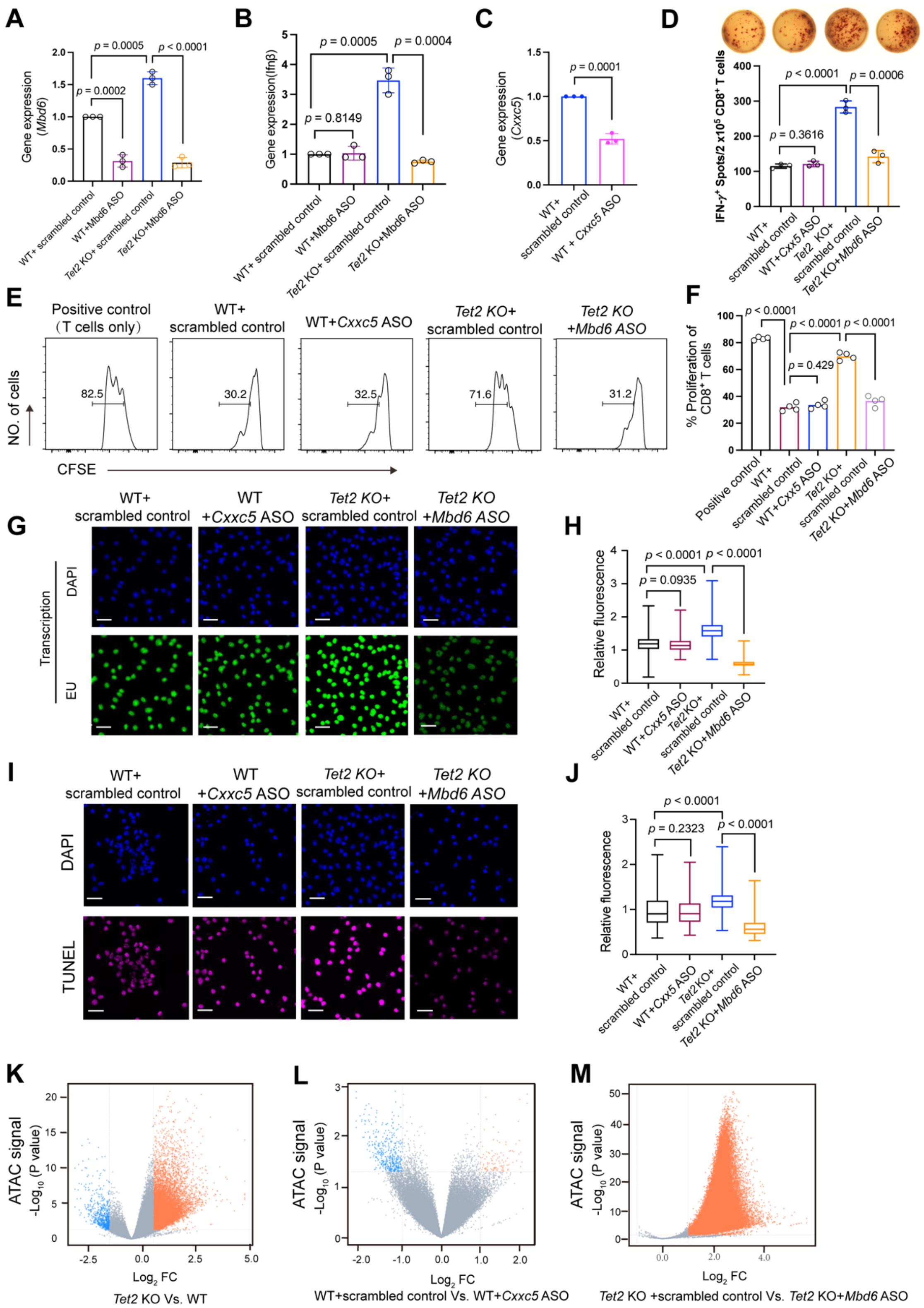

Figure S4. ***Tet2*** **regulates the chromatin remodeling in MDSCs through RNA pathway.**

(A) qPCR validation of *Mbd6* expression in the indicated groups. Data are shown as mean ± SD; each dot represents one replicate (n = 3 per group). One-way ANOVA with Bonferroni's multiple

comparisons test.

(B) qPCR validation of *Ifnb1* expression in the indicated groups. Data are shown as mean ± SD; each dot represents one replicate (n = 3 per group). One-way ANOVA with Bonferroni's multiple comparisons test.

(C) qPCR validation of *Cxxc5* knockdown efficiency. Data are shown as mean ± SD; each dot represents one replicate (n = 3 per group). Unpaired two-tailed Student's t-test.

(D) ELISPOT assay of IFN-γ positive spots secreted by $CD8^+$ T cell. Data are shown as mean ± SD; each dot represents one replicate (n = 3 per group). One-way ANOVA with Bonferroni's multiple comparison tests.

(E and F) Representative CFSE histograms (E) and quantification of $CD8^+$ T cell proliferation (F) in T cells cultured alone as a positive control or co-cultured with monocytes from the indicated groups. Data are shown as mean ± SD; each dot represents one replicate. One-way ANOVA with Bonferroni's multiple comparisons test.

(G and H) Comparisons of global nascent RNA synthesis rate among monocytes treated with indicated groups by 5-ethynyl uridine (EU) incorporation followed by fluorescence imaging (G). The nucleus was counterstained with DAPI (upper). Scale bar: 10 μm. (H) Relative fluorescence intensity of EU signal. Box plots show median, interquartile range, and minimum-to-maximum whiskers. One-way ANOVA with Bonferroni's multiple comparison tests.

(I and J) Comparisons of global chromatin accessibility among monocytes treated with indicated groups. (I) Cells were treated with DNase-TUNEL followed by fluorescence imaging (lower), The nucleus was counterstained with DAPI (upper). Scale bar: 10 μm. (J) Relative fluorescence intensity of TUNEL signal. Box plots show median, interquartile range, and minimum-to-maximum whiskers. One-way ANOVA with Bonferroni's multiple comparison tests.

(**K-M**) Volcano plot of ATAC–seq signals in *Tet2* KO versus WT monocytes (K), WT monocytes versus WT monocytes *with Cxxc5* ASO treatment (L), *Tet2* KO monocytes versus *Tet2* KO monocytes with *Mbd6* knockdown (M).

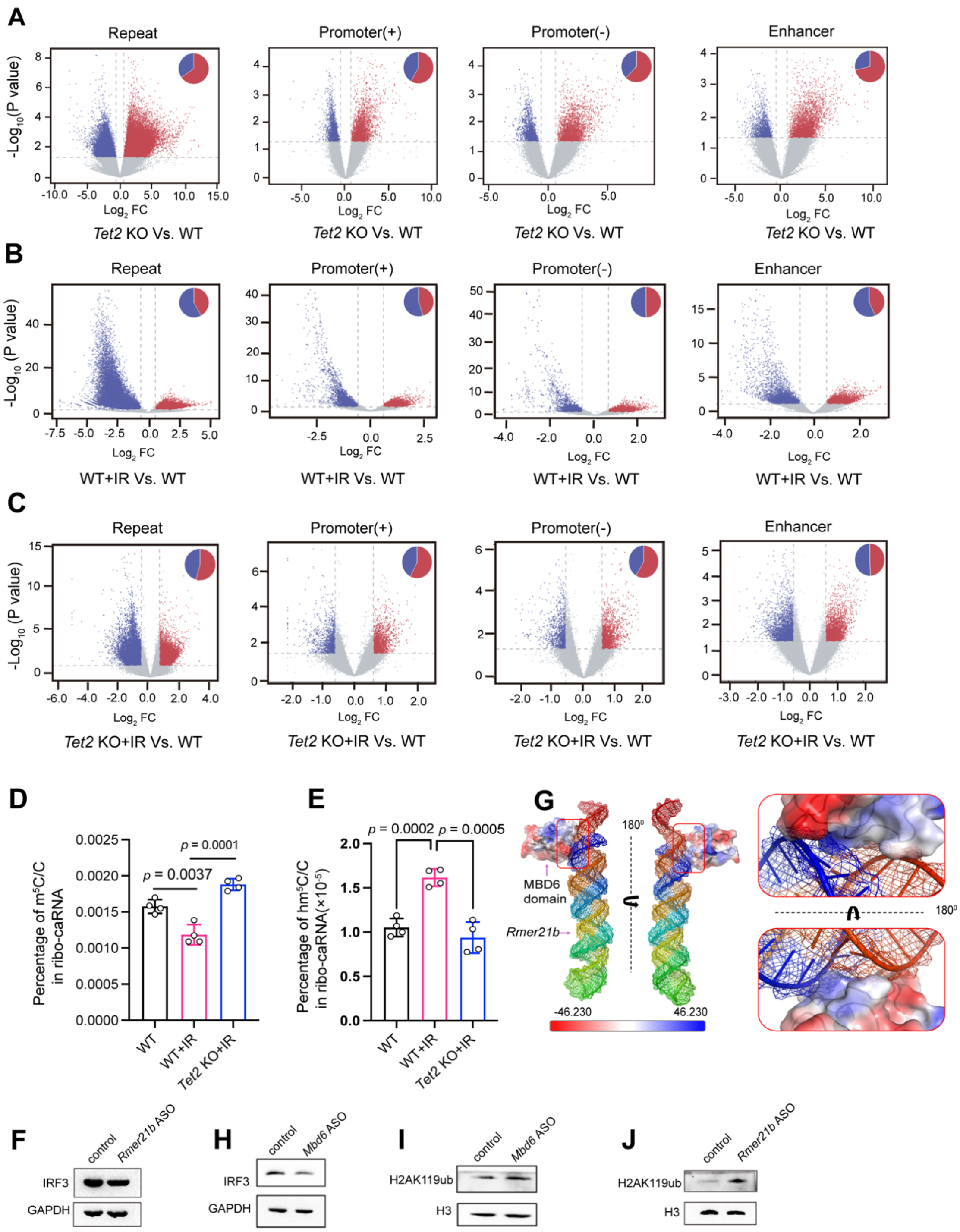

Figure S5. Differential expression analysis of chromatin-associated RNAs (caRNAs) across experimental conditions.

(A) Volcano plots displaying differential abundance of chromatin-associated RNAs (caRNAs) across experimental groups (*Tet2* KO Vs. WT), with transcripts categorized as enhancer RNAs (eRNAs), promoter-associated RNAs, and repeat RNAs.

(B) Volcano plots displaying differential abundance of chromatin-associated RNAs (caRNAs)

across experimental groups (WT+IR Vs. WT), with transcripts categorized as enhancer RNAs (eRNAs), promoter-associated RNAs, and repeat RNAs.

(C) Volcano plots displaying differential abundance of chromatin-associated RNAs (caRNAs) across experimental groups (*Tet2* KO+IR Vs. WT+IR), with transcripts categorized as enhancer RNAs (eRNAs), promoter-associated RNAs, and repeat RNAs.

(D) RNA m^5C levels in the ribominus chromatin-associated fraction were quantified as m^5C/C ratios through normalization of absolute m^5C concentrations to cytosine (C). Data are shown as mean ± SD; each dot represents one replicate (n = 4 per group). One-way ANOVA with Bonferroni's multiple comparison tests.

(E) 5-Hydroxymethylcytosine (hm^5C) levels in the ribominus chromatin-associated fraction were quantified as hm^5C/C ratios by normalizing absolute hm^5C concentrations to cytosine (C). Data are shown as mean ± SD; each dot represents one replicate (n = 4 per group). One-way ANOVA with Bonferroni's multiple comparison test.

(F) Western blot analysis of IRF3 protein levels in *Tet2* KO+IR cells treated with *Rmer21b*-targeting ASO compared to controls

(G) AlphaFold 3-predicted structural model of the MBD6 domain bound to caRNA *Rmer21b*. Left, overall views of the MBD6–*Rmer21b* complex shown before and after 180° rotation. Right, magnified views of the boxed interaction interface. The red-blue surface color scale indicates electrostatic potential, with red representing negative potential and blue representing positive potential.

(H) Western blot analysis of IRF3 protein levels in *Tet2* KO+IR cells treated with *Mbd6*-targeting ASO, compared to controls.

(I and J) Western blot analysis of H2AK119ub protein levels in *Tet2* KO+IR cells treated with *Mbd6*-targeting ASO, compared to controls (I). Western blot analysis of H2AK119ub protein levels in *Tet2* KO+IR cells treated with *Rmer21b*-targeting ASO, compared to controls (J).

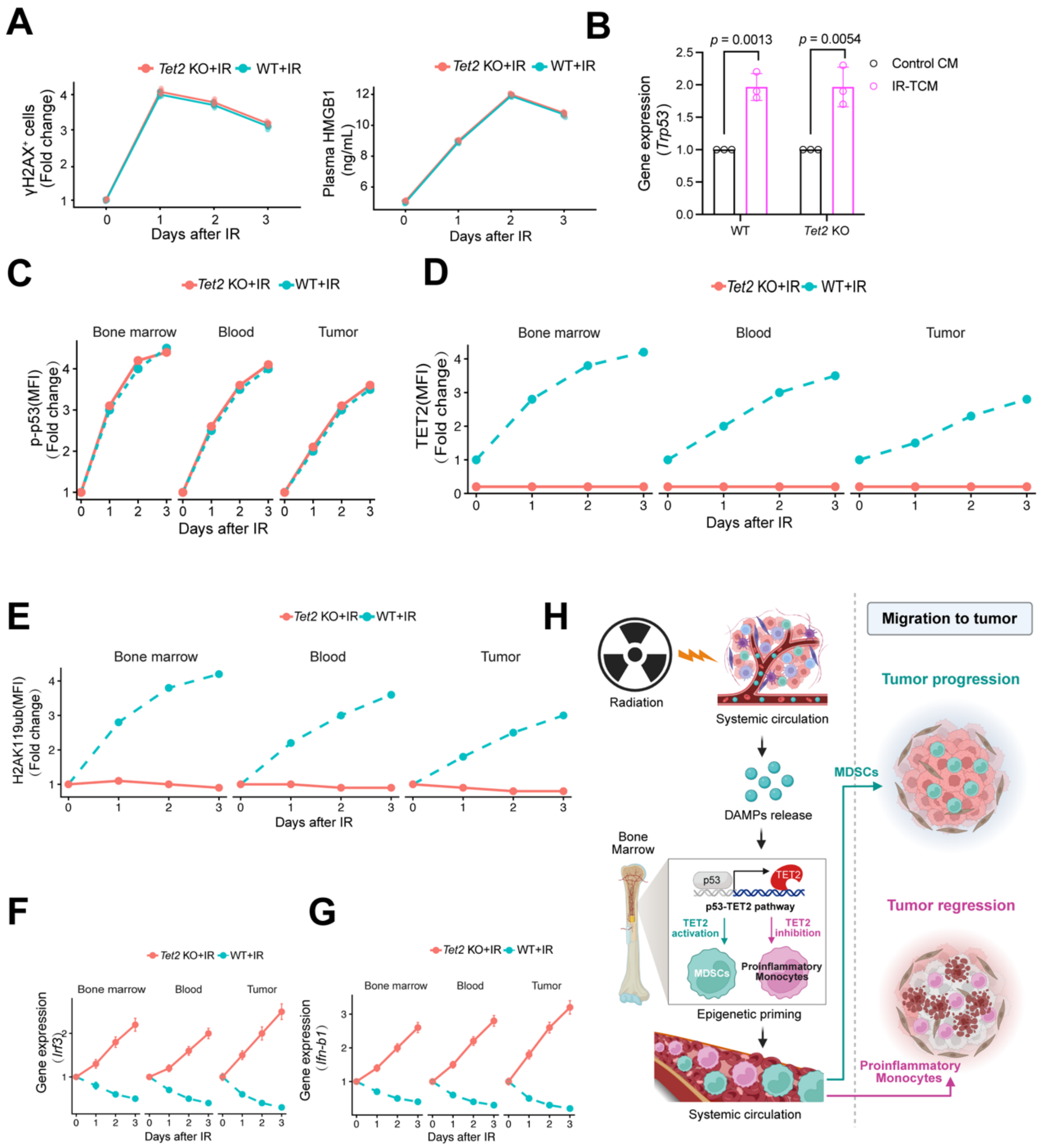

Figure S6. **Compartment-specific time-course analysis of radiation-induced myeloid responses in WT and *Tet2* cKO mice.**

(A) Time-course analysis of γH2AX signal intensity (left) and plasma HMGB1 concentration (right) in WT and *Tet2* cKO mice tumor at days 0, 1, 2, and 3 after irradiation.

(B) Relative *Trp53* mRNA expression in bone marrow monocytes isolated from WT and *Tet2* cKO mice following treatment with control conditioned medium (Control CM) or irradiated tumor-conditioned medium (IR-TCM). Data are shown as mean ± SD; each dot represents one replicate (n = 3 per group). Unpaired two-tailed Student's t-test.

(C) Time-course analysis of phosphorylated p53 (p-p53; mean fluorescence intensity [MFI] fold change) in $CD45^{+}CD11b^{+}Ly6C^{+}$ monocytes isolated from bone marrow, peripheral blood, and tumor tissues at days 0, 1, 2, and 3 after irradiation.

(D) Time-course analysis of TET2 protein levels (MFI fold change) in monocytes isolated from bone marrow, blood, and tumor compartments at the indicated time points after irradiation.

(E) Time-course analysis of H2AK119ub levels (MFI fold change) in monocytes from bone marrow, blood, and tumor tissues at days 0, 1, 2, and 3 after irradiation.

(F and G) Relative *Irf3* (F) *and Ifn-β1* (G) mRNA expression in monocytes isolated from bone marrow, blood, and tumor compartments at days 0, 1, 2, and 3 after irradiation.

(H) Schematic illustration of the experimental model showing radiation-induced systemic signaling, bone marrow epigenetic priming, and subsequent migration of myeloid cells into the tumor microenvironment.

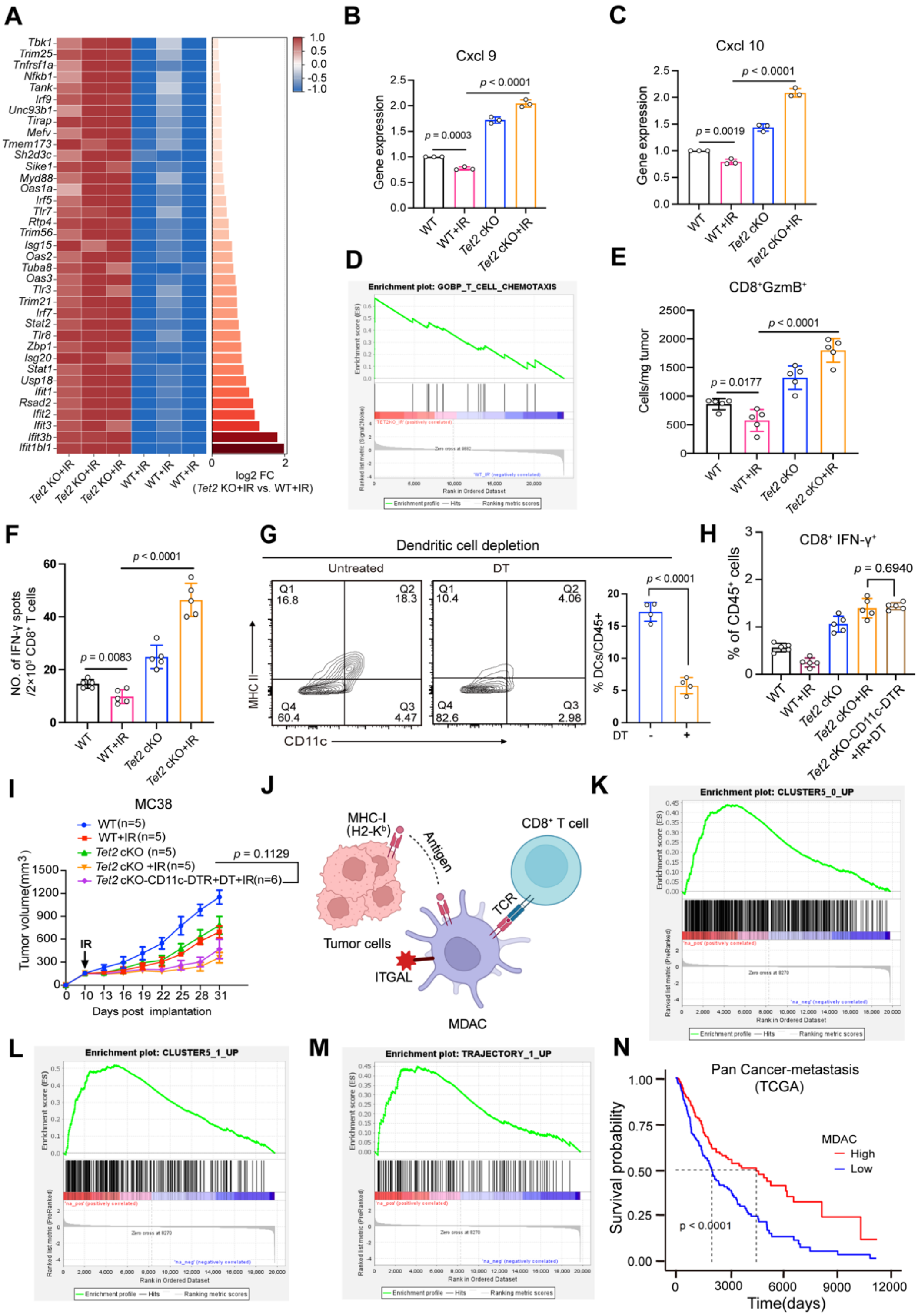

Figure S7. The pro-inflammatory myeloid-derived activating monocytes stimulate T cells for tumor elimination.

(A) RNA sequencing of CD11b$^+$Ly6C$^+$ cells revealed differential expression of interferon-regulated genes in *Tet2* cKO+IR versus WT+IR group. Red indicates higher and blue lower row-scaled

normalized expression. Right bar plot shows log_2 fold change. FDR < 0.05, DESeq2 Wald test.

(B and C) Quantitative PCR analysis of *Cxcl9* (B) and *Cxcl10* (C) expression in FACS-MDSCs from MC38 tumors at 72 hours post-IR (normalized to *Gapdh*; three technical replicates each). Data are shown as mean ± SD; each dot represents one replicate (n = 3 per group). one-way ANOVA with Bonferroni's multiple comparison tests.

(D) Gene ontology analysis of RNA-seq data identified significant enrichment of T cell chemotaxis in *Tet2* cKO+IR versus WT+IR tumors (FDR < 0.01, GSEA).

(E) The profile of tumor-infiltrating $CD8^+GzmB^+$T cells in WT or *Tet2* cKO mice three days after IR, as assessed by flow cytometry. Data are shown as mean ± SD; each dot represents one replicate (n = 5 per group). One-way ANOVA with Bonferroni's multiple comparison tests.

(F) ELISPOT analysis of IFN-γ-secreting tumor-infiltrating $CD8^+$ T cells at 72 hours post-20 Gy irradiation across the experiment groups as indicated. Data are shown as mean ± SD; each dot represents one replicate (n = 5 per group). Data were analyzed by one-way ANOVA with Bonferroni's correction.

(G) Contour plots display dendritic cell frequencies (left panel) with flow cytometric quantification (right panel) in $Lyz^{Cre}Tet2^{fl/fl}$-CD11c-DTR mice following diphtheria toxin administration. Right-panel data are mean ± SD; each dot represents one tumor (n = 4 per group). Unpaired two-sided t-test.

(H) Flow cytometric analysis of tumor-infiltrating $CD8^+IFN\text{-}\gamma^+$ T cell frequencies across experimental groups as indicated. Data are shown as mean ± SD; each dot represents one tumor (n = 4 per group). One-way ANOVA with Bonferroni's multiple comparison tests.

(I) Wild-type (*Tet2*$^{fl/fl}$)/*Tet2* cKO (Lyz^{cre}*Tet2*$^{fl/fl}$)/*Tet2* cKO-CD11c-DTR (*Lyz*^{Cre}*Tet2*$^{fl/fl}$-CD11c-DTR) mice were subcutaneously injected with ($1\times10^6/2\times10^6$) MC38 tumor cells and treated with localized tumor irradiation (20 Gy single dose) when tumors reached 100 mm^3. Diphtheria toxin was administered 24 h pre-IR and 96 h post-IR**.** Tumor growth was monitored. Data are mean ± SD; n = 5 mice per group, except *Tet2* cKO-CD11c-DTR+DT+IR, n = 6. Two-way ANOVA test with corrections for multiple variables.

(J) The Scheme. The proinflammatory MDAC can present tumor antigen to $CD8^+$ T cells and stimulate T cells for tumor elimination.

(K-M) Cross-species validation of Tet2-dependent inflammatory programs in clonal hematopoiesis.

Gene set enrichment analysis (GSEA) showing enrichment of the cluster 5_0 (K), cluster 5_1 (L),Trajectory 1 (M)–associated gene signature (derived from *Tet2*-deficient monocytes) in RNA-seq data from patients with TET2-mutant clonal hematopoiesis.

(N) Kaplan-Meier survival curves comparing overall survival between high versus low MDAC signature groups in metastasis cites of pan-cancer metastasis. Normalized gene expression and clinical data were obtained from TCGA. a two-sided log-rank Mantel-Cox test.

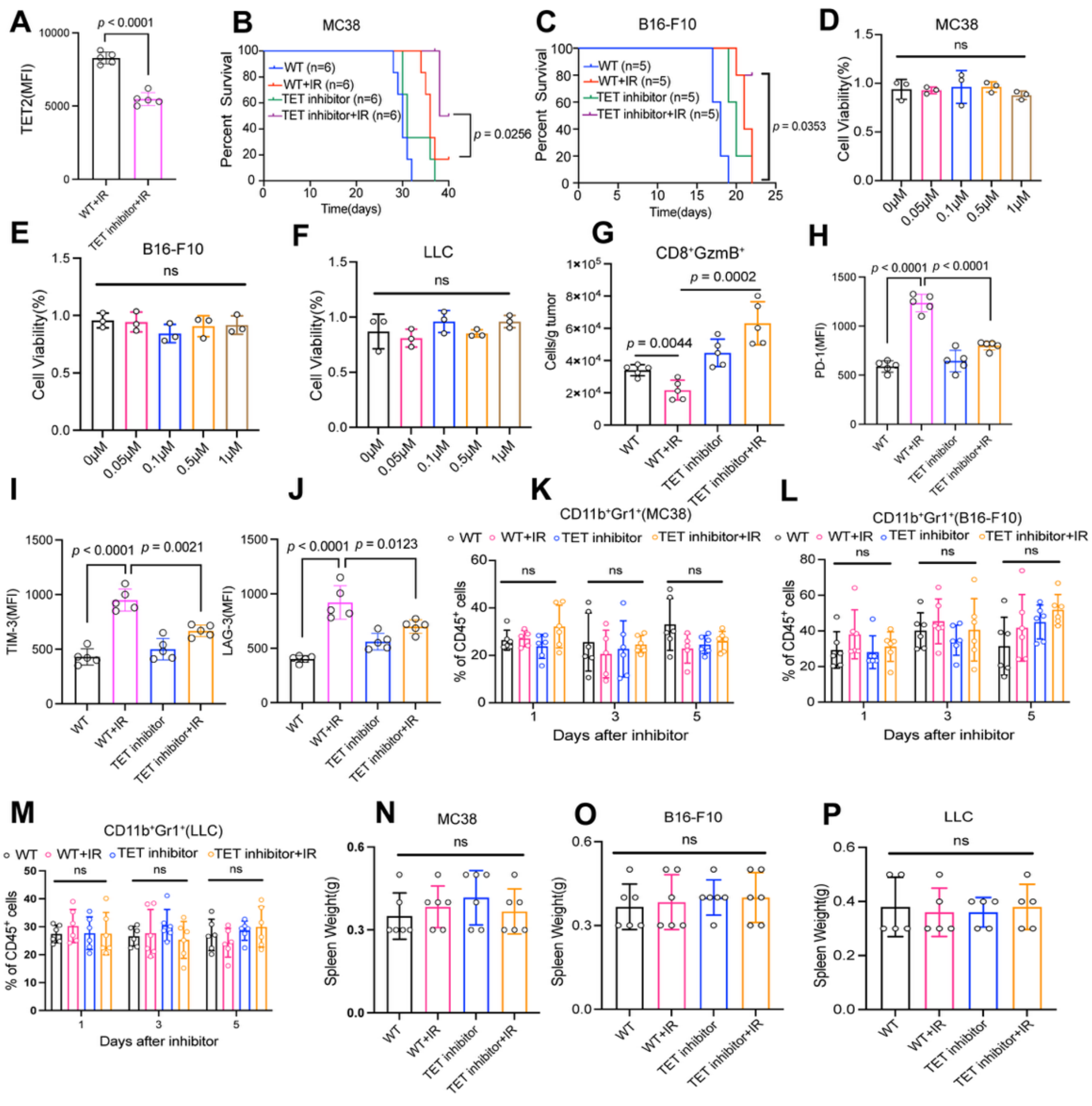

Figure S8. Pharmacological targeting of TET2 potentiates therapeutic responses to radiotherapy and immunotherapy.

(A) Flow cytometry analysis of TET2 protein expression in tumor-infiltrating $CD11b^{+}Ly6C^{+}$ monocytes on day 3 post-irradiation. Data are shown as mean ± SD; each dot represents one tumor sample. Unpaired two-tailed Student's t-test.

(B and C) Wild-type mice were injected subcutaneously with $1x10^{6}$ MC38 (B) or B16-F10 (C)

cells. When the tumor size reached 100 mm^3, tumors were treated with local IR (20 Gy, one dose). On the same day, the mice were treated with TET inhibitor (10 μM/mice, every two days for a total of five doses). Tumor survival was monitored. n = 6 mice per group in (B) and n = 5 mice per group in (C). A two-sided log-rank Mantel-Cox test.

(D-F) Cell viability of different cancer cell types (D: MC38, E: B16-F10, F: LLC) under varying concentrations of TET inhibitor. Data are shown as mean ± SD; each dot represents one replicate (n = 3 per group). One-way ANOVA with Bonferroni's multiple comparisons test.

(G) The numbers of tumor-infiltrating $CD8^+$ $GzmB^+$ in MC38 tumor-bearing mice with treatments as indicated. Data are shown as mean ± SD; each dot represents one tumor (n = 5 per group). One-way ANOVA with Bonferroni's multiple comparison tests.

(H-J) Flow cytometric analysis of checkpoint molecule expression on tumor-infiltrating $CD8^+$ T cells. Mean fluorescence intensity (MFI) of PD-1 (H), TIM-3 (I), and LAG-3 (J) is shown across treatment groups. Data are mean ± SD; each dot represents one tumor sample (n = 5 per group). One-way ANOVA with Bonferroni's multiple comparisons test.

(K-M) Time-dependent changes in $CD11b^+Gr1^+$ expression, categorized by experimental condition and tumor model (K: MC38, L: B16-F10, M: LLC). Data are mean ± SD; each dot represents one tumor sample. One-way ANOVA with Bonferroni's multiple comparisons test.

(N-P) Spleen size at the observation endpoint, categorized by experimental condition and tumor model (N: MC38, O: B16-F10, P: LLC). Data are mean ± SD; each dot represents one mouse. One-way ANOVA with Bonferroni's multiple comparisons test.
